# Analytical Model for Estimating Terrestrial Cosmic Ray Fluxes Nearly Anytime and Anywhere in the World: Extension of PARMA/EXPACS

**DOI:** 10.1371/journal.pone.0144679

**Published:** 2015-12-16

**Authors:** Tatsuhiko Sato

**Affiliations:** Research Group for Radiation Transport Analysis, Japan Atomic Energy Agency (JAEA), Shirakata 2–4, Tokai, Ibaraki 319–1195, Japan; North Shore Long Island Jewish Health System, UNITED STATES

## Abstract

By extending our previously established model, here we present a new model called “PHITS-based Analytical Radiation Model in the Atmosphere (PARMA) version 3.0,” which can instantaneously estimate terrestrial cosmic ray fluxes of neutrons, protons, ions with charge up to 28 (Ni), muons, electrons, positrons, and photons nearly anytime and anywhere in the Earth’s atmosphere. The model comprises numerous analytical functions with parameters whose numerical values were fitted to reproduce the results of the extensive air shower (EAS) simulation performed by Particle and Heavy Ion Transport code System (PHITS). The accuracy of the EAS simulation was well verified using various experimental data, while that of PARMA3.0 was confirmed by the high *R* ^2^ values of the fit. The models to be used for estimating radiation doses due to cosmic ray exposure, cosmic ray induced ionization rates, and count rates of neutron monitors were validated by investigating their capability to reproduce those quantities measured under various conditions. PARMA3.0 is available freely and is easy to use, as implemented in an open-access software program EXcel-based Program for Calculating Atmospheric Cosmic ray Spectrum (EXPACS). Because of these features, the new version of PARMA/EXPACS can be an important tool in various research fields such as geosciences, cosmic ray physics, and radiation research.

## Introduction

Galactic cosmic rays are continuously incident on the Earth, and they induce extensive air shower (EAS) by successively causing nuclear and atomic interactions in the atmosphere. Simulation of EAS over a wide energy range is essential not only for particle physics and astrophysics [1,2] but also for geosciences and radiation research. For example, evaluation of the temporal and locational variations of cosmic ray fluxes generated through EAS is very important for estimating cosmogenic nuclide yields, radiation doses for aircrews, and soft-error rates of semi-conductor devices. Furthermore, the cosmic ray-induced ionization rate in the atmosphere is one of the key quantities for determining the global temperature in the theory of cosmoclimatology [3], although its credibility has been questioned [4–6].

A number of studies were devoted for the evaluation of terrestrial cosmic ray fluxes on the basis of analytical approaches and Monte Carlo methods [7–16]. In general, Monte Carlo methods can give better estimates in comparison with analytical methods, but they require considerable computational resources. This drawback prevents the direct incorporation of Monte Carlo methods into a system for rapidly evaluating terrestrial cosmic ray fluxes at various altitudes, geomagnetic locations, and solar activities (referred to here as global conditions). The route-dose calculation code, which can automatically calculate the radiation doses suffered from a flight between certain two airports, is an example of such a system [17–19]. Modeling or database creation is required to use the results of the Monte Carlo simulations in those systems.

Considering these situations, we developed an analytical model for estimating terrestrial cosmic ray fluxes under any global condition, with the exception of altitudes higher than 20 km [20,21], by modeling the results of an EAS simulation performed using the Particle and Heavy Ion Transport Code System (PHITS) [22]. The model comprises several theoretical or empirical equations with free parameters whose numerical values were determined from the least square (LSq) fitting of EAS data. The fluxes of neutrons, protons, He ions, muons, electrons, positrons, and photons can be calculated using the model, and their applicable energy are in the range of 1 MeV–100 GeV (per nucleon for He ions), with the exception of neutrons, the fluxes of which can be calculated down to 0.01 eV. The model was designated PARMA: PHITS-based Analytical Radiation Model in the Atmosphere, and its implementation called EXcel-based Program for calculating Atmospheric Cosmic ray Spectrum (EXPACS) [23] was released to the public. PARMA and EXPACS have been extensively used in various research fields such as radiation protection [24–26], semiconductor design [27,28], and geosciences [29–33]. However, the altitude and energy ranges covered by PARMA have, on occasion, proved to be insufficient, particularly when in geosciences research. For example, estimation of cosmic ray fluxes above 20 km is important for developing a scaling model of *in situ* cosmogenic nuclide production rates. Cosmic rays below 1 MeV largely contribute to the ionization rate in the atmosphere owing to their high stopping power.

In this study, we improved PARMA by extending its applicable altitude and energy ranges up to nearly the top of the atmosphere and down to 1 keV, respectively, and designated the improved version as PARMA3.0. In the new model, the cosmic ray fluxes of heavy ions up to *Z* = 28 (Ni) were also modeled, whereas ions with *Z* > 2 were not considered in our previous study because they rarely reach altitudes below 20 km. For that purpose, the EAS simulation was conducted again using a recent version of PHITS. The procedures of the EAS simulation together with its verification results are given in Section “EAS Simulation,” details on the development of PARMA3.0 are described in the Section “Development of PARMA3.0,” and the results of verification and validation of PARMA3.0 are presented in the Section “V&V of PARMA3.0.” The final section presents our concluding remarks.

## EAS Simulation

### Simulation Procedure

The procedure for the EAS simulation in this study is basically the same as that described in our previous studies [20,21], except for the source-term determination and nuclear reaction models employed herein. The atmosphere is divided into 28 concentric spherical shells, and its maximum altitude is assumed to be 86 km. The densities of each shell are determined by referring to US Standard Atmosphere 1976. The Earth is represented as a sphere with a radius of 6378.14 km, and its composition is presumed to be the same as that of the air at sea level to obtain terrestrial cosmic ray fluxes under the ideal condition, i.e., without disturbance from the ground. The particles reaching 1000 g/cm^2^ below the ground level are discarded in the simulation to reduce the computation time. Note that the existence of soil significantly influences neutron fluxes at the ground level [8,34] due to the Earth’s albedo effect, and we used a function to convert neutron fluxes under the ideal condition to those at ground level considering water density in soil [20]. This conversion function can also be employed in this study without any modification. Thus, we ran the EAS simulation only for the idealized atmosphere in this study, otherwise the ground effect would be double counted.

In the EAS simulation, cosmic rays were incident from the top of the atmosphere assumed in the virtual Earth system, i.e., from the altitude of 86 km. Note that the atmosphere exists over 86 km, but such high altitude atmosphere has little influence on the EAS simulation. The GCR protons and heavy ions with energies and charges up to 1 TeV/n and 28 (Ni), respectively, were considered as the source particles. The GCR fluxes at 1 astronomical unit (1 AU, around the Earth) can be estimated from their local interstellar (LIS) fluxes considering the modulation due to the solar wind magnetic field, so-called solar modulation. In this study, the model recently proposed by Matthiä et al. [35] was employed for calculating the GCR fluxes at 1 AU because of its simplicity and accuracy compared with our original model, which was used in the previous study.

In the Matthiä model, the energy-differential GCR fluxes at 1 AU for particle type *i* with energy *E* for solar modulation index *W*, *ϕ*_*i*,1AU_(*E*,*W*), can be calculated on the basis of force field formalism [36] as follows:

ϕi,1AU(E,W)=a1,iβa2,iR(E)a3,i{R(E)R(E)+[0.37+0.0003W1.45]}a4W+a5Ai|Zi|1β, (1)

where *A*_*i*_ and *Z*_*i*_ are the mass and charge numbers of the particle, respectively, *R*(*E*) is the rigidity of the particle in GV, and *β* is the speed of the particle relative to that of light. *W* is generally calculated from the sun spot number; however, in the Matthiä model, it is determined from cosmic ray measurements and neutron monitor count rates, as described later. The parameters *a*_1,*i*_ to *a*_3,*i*_ are free parameters depending on particle type *i*, while *a*_4_ and *a*_5_ are constant for all particles. The numerical values of these parameters are listed in Ref. [35]. The EAS simulations were conducted for five solar activities *W* = 0, 50, 100, 150, and 200, and 21 geomagnetic fields with the vertical cut-off rigidities, *r*_c_, from 0 to 20 GV. The azimuth and zenith dependences of the cut-off rigidity were considered by assuming that geomagnetism can be expressed simply using a dipole magnet, as described in Ref. [8].

The atmospheric propagation of the incident cosmic rays and their associated EAS was simulated using PHITS version 2.73. The code can deal with the transport of nearly all particles up to 1 TeV or 1 TeV/n, except for electrons and positrons, whose maximum energies were limited to 10 GeV. In the EAS simulation performed in our previous study, the Japanese Evaluated Nuclear Data Library/High Energy file (JENDL/HE) [37] was employed for simulating neutron-induced nuclear reactions below 3 GeV. JENDL/HE includes differential cross sections of neutrons and protons up to 3 GeV, which are generally more accurate than those obtained from conventional nuclear reaction models. However, JENDL/HE cannot be used in combination with the event generator mode [38], which must be used in this study because the determination of charged particles emitted from neutron-induced reactions below 20 MeV is indispensable for calculating their fluxes below 1 MeV. Instead, we employed the default model and data library of PHITS 2.73, which are Intra-Nuclear Cascade of Liège (INCL) [39] and JENDL-4.0 [40], respectively, for simulating the neutron reactions above and below 20 MeV, respectively. The total nucleon–nucleus interaction cross sections were calculated using a model particularly adjusted for high-energy particle transport simulations [41].

The EAS simulation took approximately two months using a parallel computer with 64 CPUs. In the simulation, all particles were traced down to 10 keV, with the exception of neutrons, which were transported down to 0.01 eV. The variance-reduction technique was adopted for transporting low-energy electrons, positrons, and photons to reduce the computational time. The fluences of all simulated particles for each spherical shell of the atmosphere were scored as a function of their energy using the track-length tally in PHITS. For verification, their vertical fluxes were also estimated from the simulation using the surface-crossing tally.

### Simulation Results

**Fig 1:**
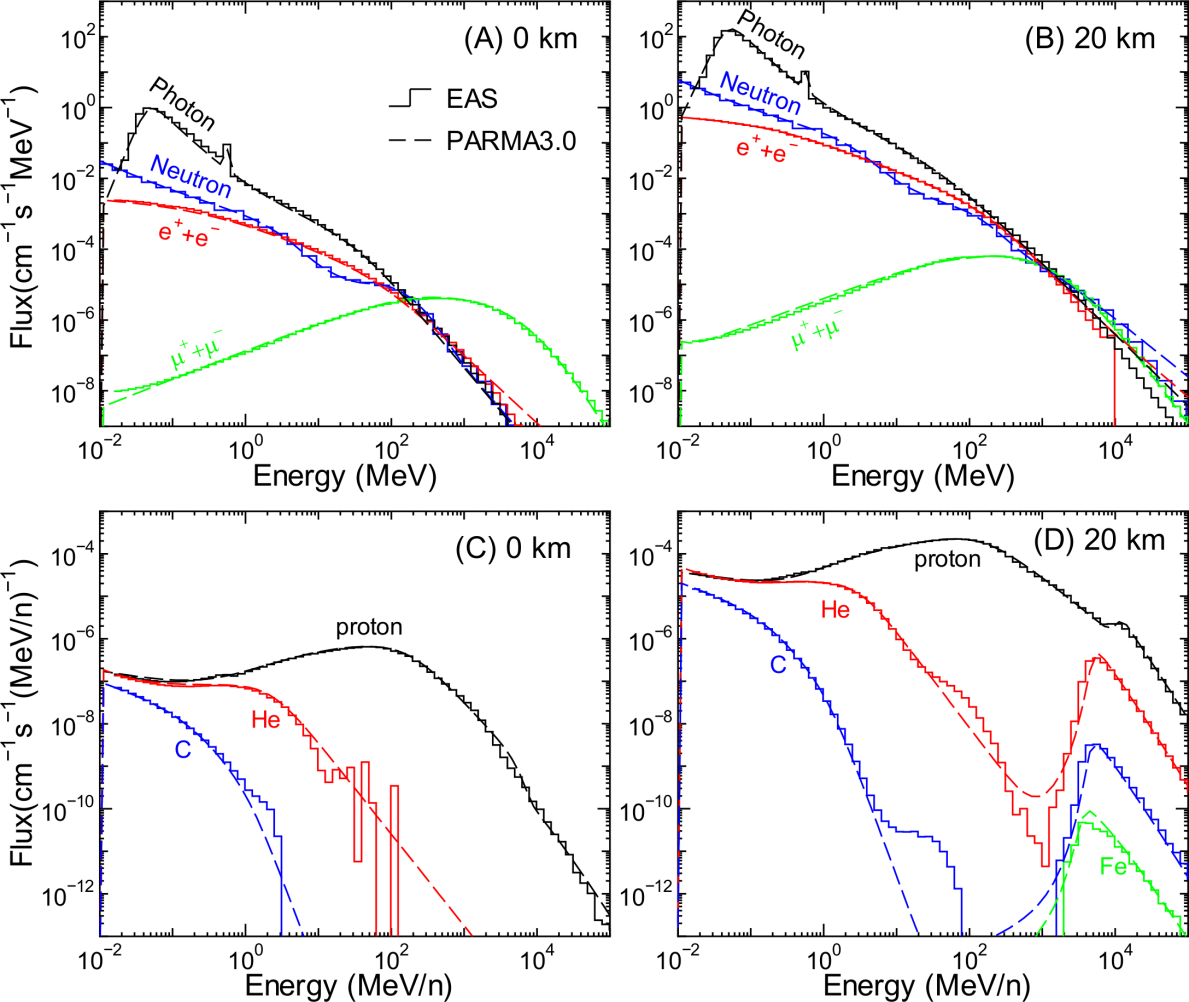
Examples of calculated cosmic ray fluxes obtained from EAS simulation and PARMA3.0. The upper panels show the fluxes of neutrons, photons, muons, and the sum of the fluxes of electrons and positrons, while the lower panels show those of protons, He, C, and Fe ions. The left and right panels show the data at altitudes of 0 and 20 km, respectively. The solar and geomagnetic conditions are fixed at *W* = 0 and *r*_c_ = 12 GV, respectively, for all panels.

As examples of the EAS simulation results, the calculated cosmic ray fluxes of protons, He, C, and Fe ions, neutrons, muons, photons, electrons, and positrons at altitudes of 0 and 20 km are shown in Fig 1. The solar and geomagnetic conditions of those data are for *W* = 0 and *r*_c_ = 12 GV, which correspond to the solar minimum condition around Tokyo, Japan. The statistical uncertainties of those data are generally small—less than 5%—except for those of high-energy ion data. Furthermore, the corresponding data calculated by the analytical model proposed in the next section, PARMA3.0, are plotted in the figure.

As is well known, the calculated fluxes at 20 km are much higher than those at 0 km, with the exception of those of muons. Ion spectra at 20 km comprise high- and low-energy components, which are attributed to primary cosmic rays and their secondary particles, respectively. In contrast, high-energy heavy ions do not exist at 0 km because primary cosmic rays cannot penetrate to the sea level. The small peaks observed in the low-energy photon spectra can be attributed to the 511 keV γ-rays produced by electron–positron annihilation.

**Fig 2:**
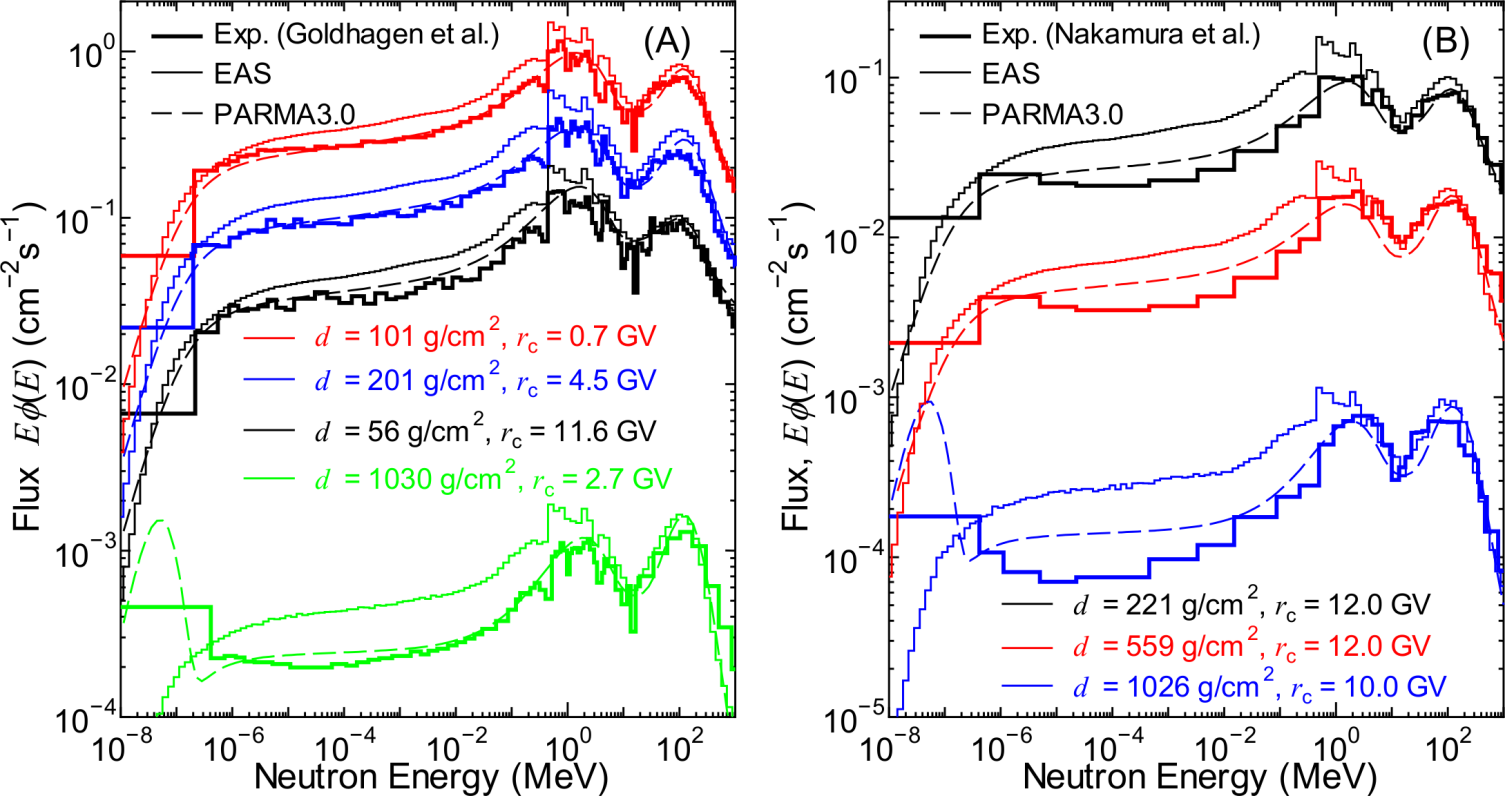
Neutron fluxes measured in aircraft as well as on ground [42,43] in comparison with corresponding calculated data obtained from EAS simulation and PARMA3.0.

To examine the accuracy of the EAS simulation, the calculated neutron fluxes were compared with the experimental data measured in aircraft and on ground [42,43]. The results of the comparison are shown in Fig 2, together with the corresponding data calculated using PARMA3.0. It should be mentioned that the neutron fluxes are generally depicted by their energy-weighted values, *Eϕ*(*E*), with the unit cm^−2^s^−1^ in this study to cover the wide energy range to be shown. It is evident from the graphs that the neutron fluxes obtained from the EAS simulation agree fairly well with the experimental data, although slight overestimations were observed in the lower-energy fluxes, particularly ground-level data. These overestimations are attributed to the ignorance of the ground or aircraft effect in the EAS simulation, because hydrogen-rich materials such as water in ground and fuel in aircraft effectively reduce the low-energy neutron fluxes except for thermal energies around 0.025 eV, as discussed in [20]. In contrast, the influences of ground or aircraft on the neutron fluxes can be considered in our analytical model, and hence, PARMA3.0 can reproduce the experimental data more precisely. Particularly, the thermal-energy peaks observed at the ground level are reproduced only by PARMA3.0 because they are formed by albedo neutrons.

**Fig 3:**
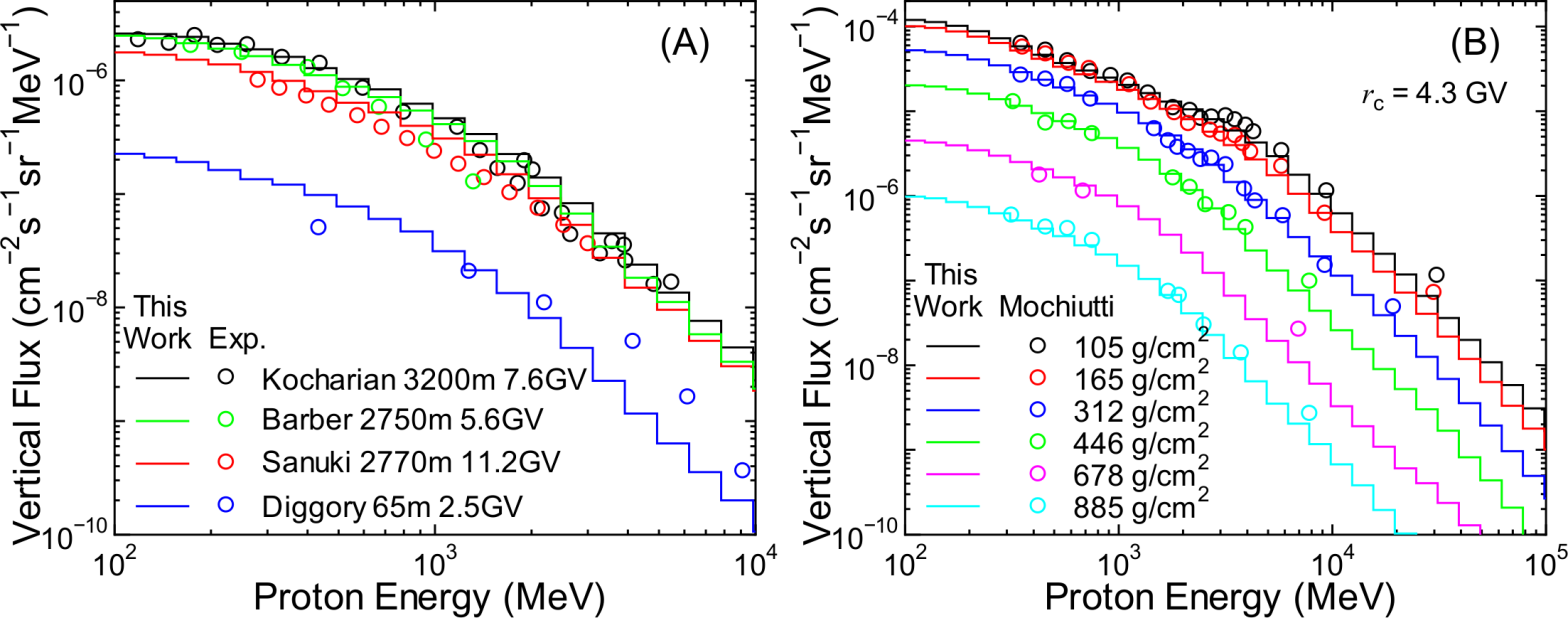
Vertical proton fluxes measured by various authors [44–48] in comparison with EAS data.

**Fig 4:**
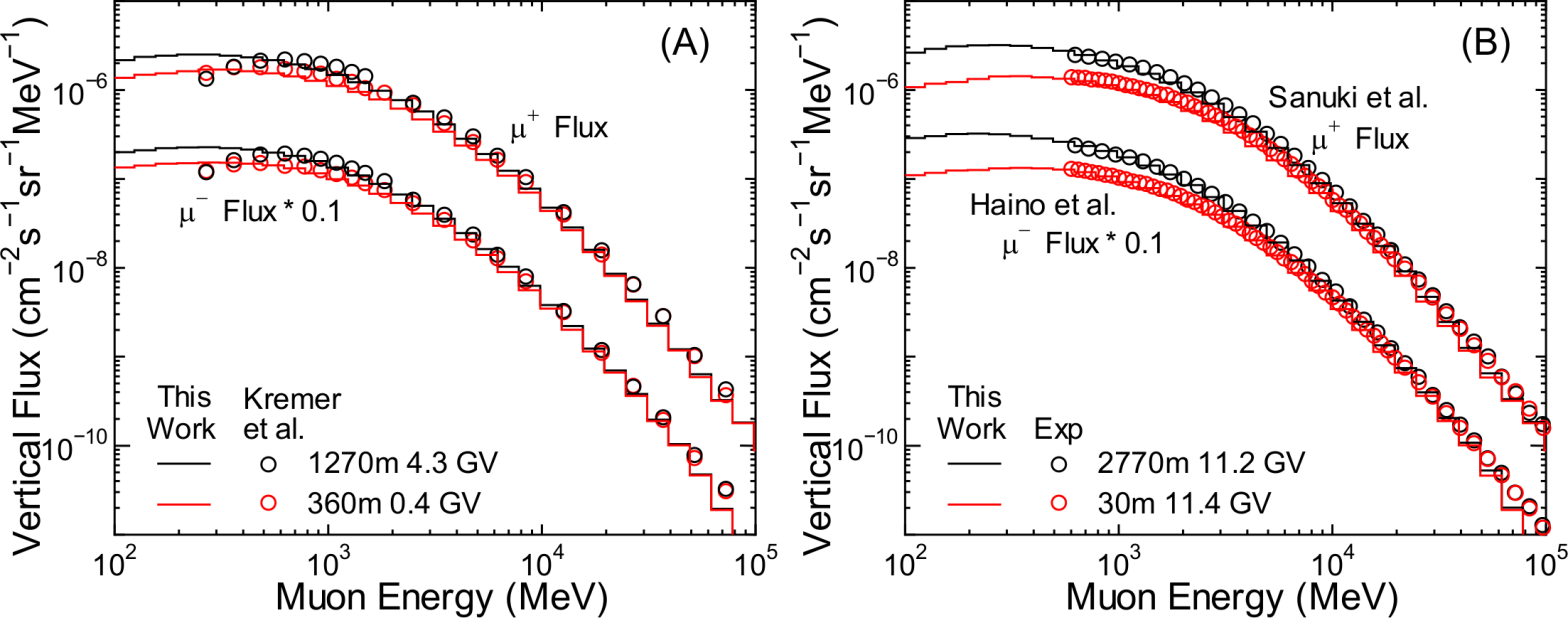
Vertical muon fluxes measured by various authors [49–51] in comparison with EAS data.

**Fig 5:**
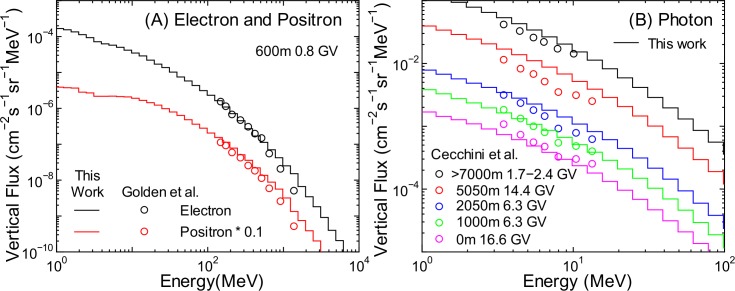
Vertical electron, positron, and photon fluxes measured by Golden et al. [52] and Cecchini et al. [53] in comparison with EAS data.

To verify the accuracy of calculated fluxes of other particles, it is ideal to benchmark the EAS simulation results using angular integrated fluxes, as in the case of neutrons. However, doing so is not feasible because of the scarcity of relevant experimental data. Therefore, we compared the vertical fluxes obtained from the EAS simulation with the corresponding experimental data of protons [44–48], muons [49–51], electrons, positrons [52], and photons [53], as shown in Figs 3–5. It is evident from the graphs that the calculated vertical fluxes agree well with the measured data, with the exception of photons, thus indicating the reliability of the EAS simulation. The calculated photon fluxes are greater than the measured data at higher altitudes, while the reverse is true at sea level. The reasons underlying these disagreements are currently under investigation. Slight underestimation of the muon fluxes over 10 GeV is attributed to excluding incident cosmic rays over 1 TeV/n; however, this discrepancy does cause underestimation of the muon fluxes when using PARMA3.0, as discussed in the next section.

## Development of PARMA3.0

### General Description of PARMA3.0

PARMA3.0 (PHITS-based Analytical Radiation Model in the Atmosphere, Version 3.0) facilitates instantaneous estimation of the terrestrial cosmic ray fluxes of neutrons, protons, ions with charge up to 28 (Ni), muons, electrons, positrons, and photons nearly anytime and anywhere in the Earth’s atmosphere. The unit of the output fluxes is cm^−2^s^−1^MeV^−1^, supplying the atmospheric depth in g/cm^2^, cut-off rigidity in GV, the solar modulation index *W*, and kinetic energy in MeV, with the exception of ions, where the kinetic energy is given in MeV/n to facilitate estimation of their fluxes in cm^−2^s^−1^(MeV/n)^−1^.The particle energies covered by PARMA3.0 are 10 keV–1 TeV (per nucleon for ions), except for neutrons, whose fluxes can be calculated down to 0.01 eV. The model comprises numerous analytical functions with parameters whose numerical values were determined by LSq fitting to the EAS data.

The global conditions of the EAS data considered in the LSq fitting are atmospheric depth of 0.15–1024 g/cm^2^, cut-off rigidity of 0–20 GV, *W* index of 0–200. In practical use of PARMA3.0, these ranges can be slightly extended by extrapolating the data. Thus, the applicable ranges of PARMA3.0 cover from the sea level to the top of the atmosphere in terms of altitude, from the polar region to the equatorial region in terms of geomagnetism, and from the minimum to the maximum solar activities recorded in the last 400 years. It should be noted that the EAS simulation results for *W* = 0 and 150 were considered as the data corresponding to minimum and maximum solar activity conditions in the development of PARMA3.0. The data of the other *W* index were used only for determining the solar-modulation dependence of secondary particle fluxes, as discussed later in this section.

### Development Strategy

The forms of analytical functions used in PARMA3.0 are similar to those proposed in our previous study, although the model development strategies are different. In our previous study, we attempted to reproduce the cosmic ray fluxes obtained from the EAS simulation as simply as possible, using mostly analytical functions having some physical meaning. In contrast, in this study, we aimed to reproduce the EAS simulation results as precisely as possible over wider altitude and energy ranges. Thus, we introduced several correction factors to estimate the cosmic ray fluxes at higher altitudes or lower energies, which were omitted in our previous study. Little physical meaning exists in the forms of the equations used to calculate those correction factors.

Furthermore, altitude dependences of some free parameters used in PARMA3.0 are so complex that they cannot be expressed by a simple form over a wide altitude range—from sea level to the top of the atmosphere. Thus, we evaluated the numerical values of those parameters only for discrete altitude levels for which the EAS data were available and determined the values for intermediate altitudes by simply interpolating the evaluated data. Consequently, the number of parameters evaluated in this study became numerous in comparison to those in our previous study. Note that the evaluated parameters are not tabulated in this paper, and they are provided in the new version of EXPACS, which is available from its website [23].

### Proton and Ion Fluxes

**Fig 6:**
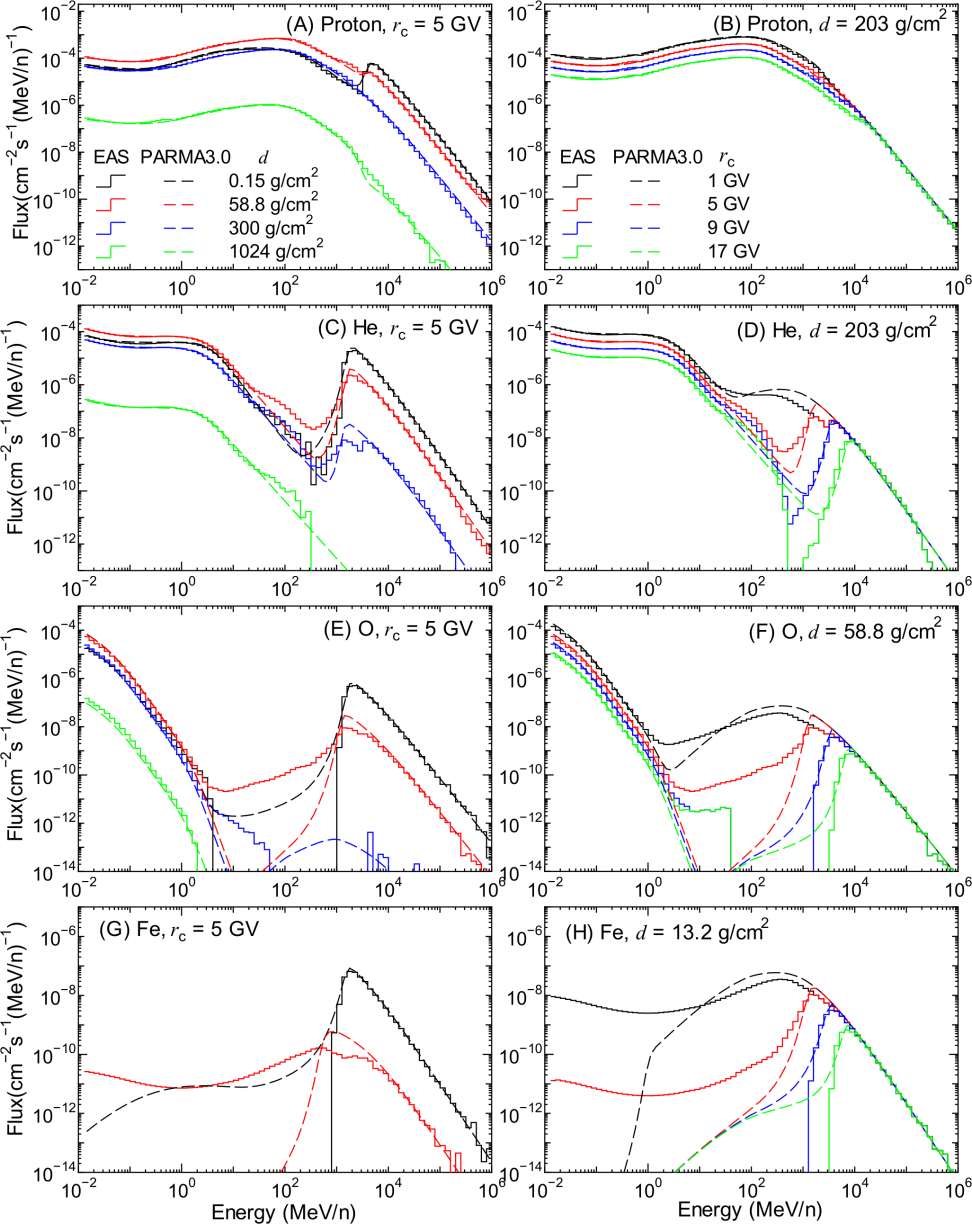
Proton, He-, O-, and Fe-ion fluxes obtained from EAS simulation and PARMA3.0. The left and right panels indicate the atmospheric depth and the vertical cut-off rigidity dependences of the fluxes, respectively.

Fig 6 shows the proton, He-, O-, and Fe-ion fluxes obtained in the EAS simulation in comparison with the corresponding data calculated using PARMA3.0. The left and right panels show the atmospheric depth and the vertical cut-off rigidity dependences of the fluxes, respectively. As can be seen in the figure, their spectra can be divided into higher- and lower-energy components, although they are not clearly distinguished in the case of protons, and only high-energy components exist in the case of Fe ions. The two components predominantly comprise primary cosmic rays and their secondary particles produced in the atmosphere, respectively. The switching energy between the two components depends on the cut-off rigidity, as discussed later in this section. In the same manner as our previous study, we first proposed mathematical functions to estimate the primary and secondary particle fluxes separately, and then developed a model for calculating the total fluxes by combining them.

#### Primary cosmic ray fluxes in atmosphere

Considering the energy loss and nuclear interactions in the atmosphere, we assumed that the primary cosmic ray fluxes of particle *i* with energy *E* for solar modulation index *W* at atmospheric depth *d*, *ϕ*_*i*,pri_(*E*,*d*,*W*), can be expressed as follows:

$$\begin{array}{l} \phi_{i,\text{pri}}\left(E,d,W\right)=\phi_{i,1\text{AU}}\left(E+S_{i}\left(E\right)d,W\right)\left(4\pi -\Omega_{\text{E}}\right)\\ \;\times \left\{b_{1,i}\left(d\right)\text{exp}\left(-b_{2,i}d\right)+\left[1-b_{1,i}\left(d\right)\right]\text{exp}\left(-b_{3,i}d\right)\right\}, \end{array}$$ (2)

where *ϕ*_*i*,1AU_ is the primary cosmic ray flux at 1 AU, as calculated using Eq (1), and its unit is cm^−2^s^−1^(MeV/n)^−1^sr^−1^. *S*_*i*_(*E*) is the stopping power of particle *i* with energy *E* in the atmosphere, and its unit is (MeV/n)(g/cm^2^)^−1^. *Ω*_E_ is the solid angle of the Earth from a point at the top of the atmosphere in our EAS simulation, and *b*_1,*i*_–*b*_3,*i*_ are free parameters depending on particle type *i* as well as *d* in the case of *b*_1_. The value of *E* + *S*_*i*_(*E*)*d* indicates the back-calculated primary cosmic ray energy at 1 AU under the assumptions that the stopping power is constant at higher energies and the cosmic ray is vertically incident on the atmosphere. The second assumption is inadequate particularly for higher altitudes, and the inaccuracy induced by this supposition was compensated by introducing altitude dependence into parameter *b*_1,*i*_, which was regarded as a constant in our previous study. The effect of the magnetosphere was not considered in this equation, and it will be considered in the equation for combining the primary fluxes with the secondary ones, i.e., in Eq (8) proposed later in this section.

The numerical values of *b*_1,*i*_–*b*_3,*i*_ were determined from the LSq fitting to the EAS data only for high energies and low cut-off rigidities because Eq (2) was introduced to calculate the primary cosmic-ray fluxes without considering the magnetosphere; the low-energy and high cut-off rigidity data were disturbed by the contributions of secondary particles and the effect of the magnetosphere, respectively. The value of *S*_*i*_(*E*) calculated using PHITS were adopted in the LSq fitting. Because of large statistical uncertainties of the EAS data for non-abundant ion species such as Ni, particles were categorized into six groups according to their charge: *Z* = 1, 2, 3–5, 6–9, 10–19, and 20–28, and the values of the *b* parameters were assumed to be the same within each category.

#### Secondary cosmic ray fluxes in atmosphere

In the PHITS simulation, there are 3 types of reaction mechanisms for producing secondary particles, which are the intra-nuclear cascade, evaporation, and recoil processes. The intra-nuclear cascade process can emit secondary particles with energy equivalent to that of the incident particle, while the other two processes emit only low-energy particles with energies below approximately 20 MeV. Only protons and He ions were generated through the cascade process in the PHITS simulation, although the maximum energy of secondary He ions was limited to approximately 300 MeV/n. Other ions were produced only by the recoil and evaporation processes, but the production yields of ions heavier than oxygen were negligibly small, as shown in panels (G) and (H) of Fig 6 because the atmosphere predominantly comprises nitrogen and oxygen atoms. Therefore, we modeled the secondary ion fluxes with charges of up to 8 in this study.

Our previous study suggested that the relative shapes of the secondary particle fluxes are almost independent of the cut-off rigidity and solar activity for protons, and even of any global conditions in the case of He ions. This was because the spectra of the particles produced by the recoil and evaporation processes are nearly insensitive to reaction conditions such as incident particle type and energy, and secondary He ions were assumed to be generated only through those processes in our former EAS simulation. In the EAS simulation in this study, secondary He ions were produced also by the intra-nuclear cascade process in the same manner as that for protons. Thus, we propose the following functions to express the secondary ion fluxes *ϕ*_*i*,sec_(*E*, *d*, *r*_c_, *W*):

$$\begin{array}{l} \phi_{i,\text{sec}}\left(E,d,r_{\text{c}},W\right)=\Phi_{i}\left(d,r_{\text{c}},W\right)\varphi_{i,\text{all}}\left(E,d\right)\;\text{for Z}\leq 2,\\ \;=\Phi_{i}\left(d,r_{\text{c}},W\right)\varphi_{i,\text{eva}+\text{rec}}\left(E\right)\;\text{for}\;3\leq \text{Z}\leq 8,\\ \;=0\;\text{for}\;9\leq \text{Z}, \end{array}$$ (3)

where *Φ*_*i*_(*d*,*r*_c_,*W*) is the normalization flux in cm^−2^s^−1^, and *φ*_*i*,*all*_(*E*,*d*) and *φ*_*i*,*eva+rec*_(*E*) are the normalized energy spectra of the secondary particles *i* in (MeV/n)^−1^, which are produced by all three reaction mechanisms and only by the evaporation and recoil processes, respectively. The flux of particle *i* at 1 MeV/n was selected as the numerical value of *Φ*_*i*_ because the contributions of the primary particles are almost negligible for such a low energy level. Note that the symbols *ϕ*, *φ*, and *Φ* are used for representing absolute fluxes, normalized energy spectra, and fluxes used for normalization, respectively, throughout this paper.

**Fig 7:**
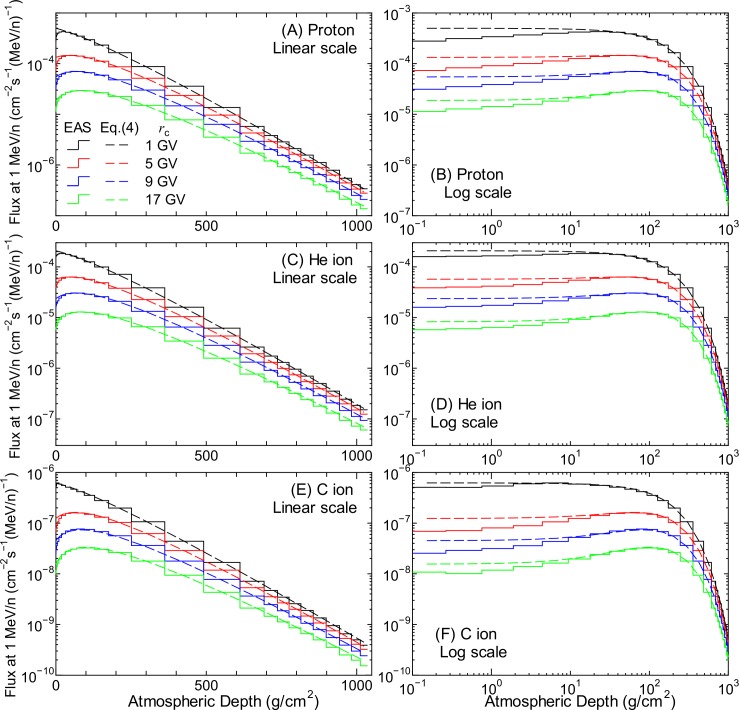
Fluxes of protons, He, and C ions at 1 MeV/n as obtained from EAS simulation and the corresponding normalization fluxes calculated using Eq (4). The left and right panels show the same data drawn along different x-axes, in particular, the linear and logarithmic scales of atmospheric depth, respectively.

Fig 7 shows proton, He-, and C-ion fluxes at 1 MeV/n, as obtained from the EAS simulation, and these values should be reproduced by LSq fitting of *Φ*_*i*_. The left and right panels show the same data drawn along different x-axes, in particular, the linear and logarithmic scales of atmospheric depth, respectively. In the same manner as our previous study, the atmospheric-depth dependence of the normalization fluxes for the solar minimum and maximum conditions, $\Phi_{i}^{\left(W\mp \right)}$, is expressed as follows:

$$\Phi_{i}^{\left(W\mp \right)}(d,r_{\text{c}})=c_{1,i}^{\left(W\mp \right)}(r_{\text{c}})\left\{\exp\left[-c_{2,i}^{\left(W\mp \right)}(r_{\text{c}})d\right]-c_{3,i}^{\left(W\mp \right)}\text{exp}\left[-c_{4,i}^{\left(W\mp \right)}(r_{\text{c}})d\right]\right\},$$ (4)

where $c_{1,i}^{\left(W\mp \right)}$–$c_{4,i}^{\left(W\mp \right)}$ are free parameters depending on the cut-off rigidity *r*_c_. Note that the superscript (*W*∓) indicates that the variable is defined respectively for the solar minimum and maximum conditions. The derivation of this equation was described in detail in our previous paper [20].

**Fig 8:**
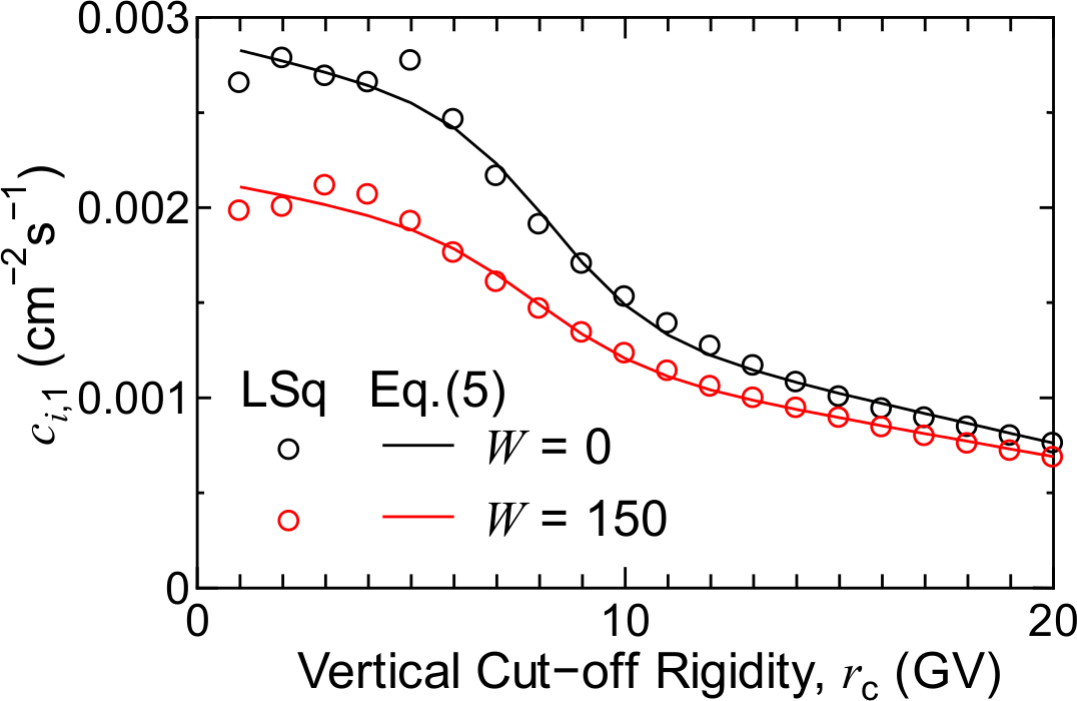
Numerical values of $c_{1,i}^{\left(W\mp \right)}$ of protons. Open circles denote the data obtained directly from the fitting of LSq to the fluxes at 1 MeV/n, while the solid lines represent the corresponding data calculated using Eq (5).

We determined the values of $c_{1,i}^{\left(W\mp \right)}$–$c_{4,i}^{\left(W\mp \right)}$ by LSq fitting to reproduce the fluxes at 1 MeV/n obtained in the EAS simulation. As examples, the evaluated $c_{1,i}^{\left(W\mp \right)}$ of protons are shown in Fig 8. The dependence of the evaluated *c* parameters on *r*_c_ was expressed by the sum of sigmoid and linear functions as follows:

$$c_{j,i}^{\left(W\mp \right)}\left(r_{\text{c}}\right)=g_{j1,i}^{\left(W\mp \right)}+g_{j2,i}^{\left(W\mp \right)}r_{\text{c}}+\frac{g_{j3,i}^{\left(W\mp \right)}}{1+\text{exp}\left\{\left[r_{\text{c}}-g_{j4,i}^{\left(W\mp \right)}\right]/g_{j5,i}^{\left(W\mp \right)}\right\}},$$ (5)

where $g_{j1,i}^{\left(W\mp \right)}$–$g_{j5,i}^{\left(W\mp \right)}$ are free parameters, whose numerical values were determined by LSq fitting to the evaluated $c_{j,i}^{\left(W\mp \right)}$ parameters. The results of the fitting are also plotted in Fig 8. The agreement between the evaluated $c_{j,i}^{\left(W\mp \right)}$ parameters and the corresponding data obtained from Eq (5) indicates the adequacy of the form of the equation. Note that the same function form as Eq (5) was employed to express the *r*_c_ dependences of all parameters introduced in this study. It should be also mentioned that the numerical values of the *c* parameters for *r*_c_ < 1 GV were assumed to be same as those at *r*_c_ = 1 GV because secondary particle fluxes are nearly insensitive to the cut-off rigidity for such low values.

The normalization fluxes obtained from Eq (4) by substituting the *c* parameters calculated using Eq (5) are also drawn in Fig 7. It is evident from the figure that the calculated normalization fluxes agree well with the flux at 1 MeV/n obtained from the EAS simulation, with the exception of those at atmospheric depths lower than approximately 20 g/cm^2^, i.e., altitudes higher than 27 km. For such high altitudes, Eq (4) slightly overestimates the simulation results. Similar tendencies are also observed for other ions and for the solar maximum condition, although they are not shown in the figure. For calculating the normalization fluxes under arbitrary solar conditions, we used the same functions that were employed in our previous study (see Eqs (8)–(10) in Ref. [21]). Note that the solar activity was expressed by the force field potential in GV in those equations, which can be deduced from the *W* index as 0.37 + 0.0003 *W* ^1.45^, according to Eq (1).

**Fig 9:**
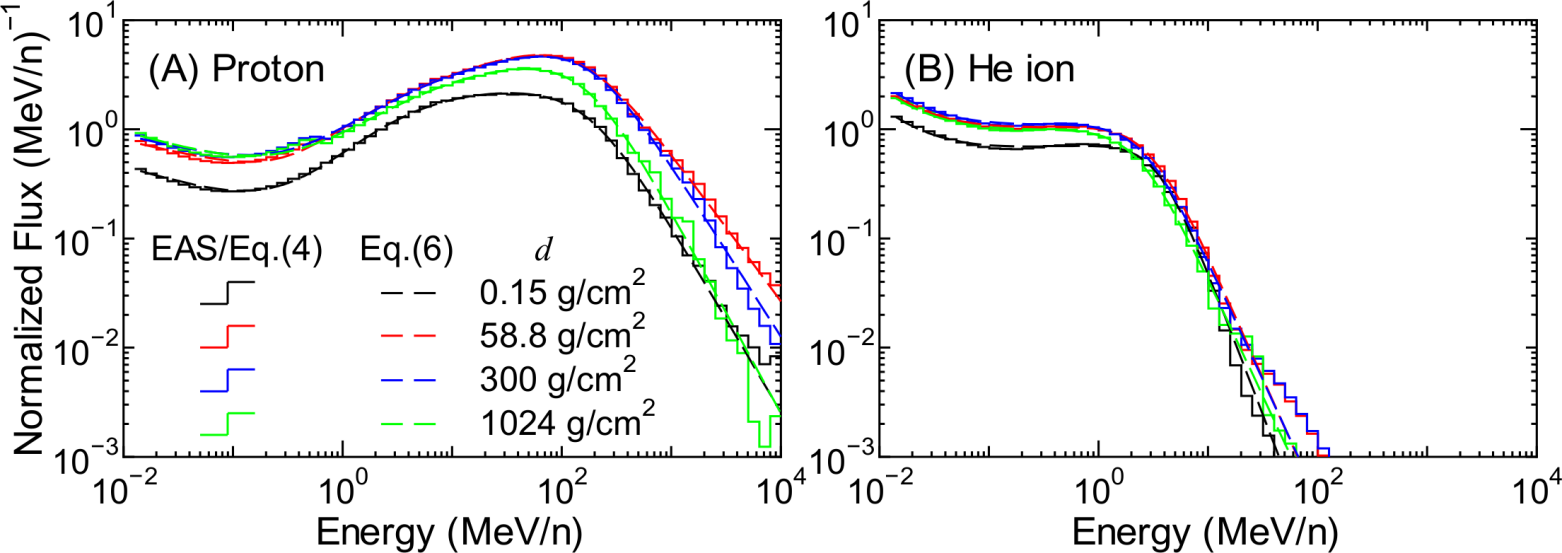
Normalized spectra of (A) protons and (B) He ions for each atmospheric depth.

**Fig 10:**
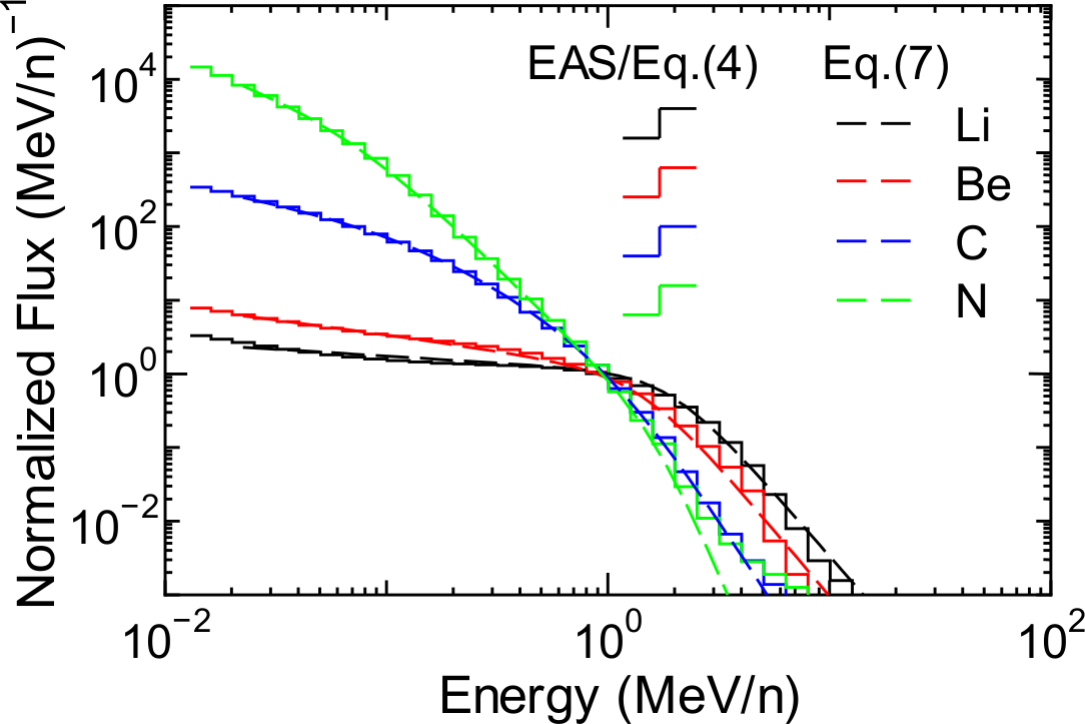
Normalized spectra of heavy ions averaged over all global conditions.

To determine *φ*_*i*,*all*_(*E*,*d*) and *φ*_*i*,*eva+rec*_(*E*) in Eq (3), we evaluated the normalized particle spectra, which are the ratios of the fluxes obtained from the EAS simulation to *Φ*_*i*_ calculated using Eq (4), as a function of the atmospheric depth for protons and He ions, and averaged over all global conditions for heavier ions. Examples of the normalized spectra are shown in Fig 9 for protons and He ions, and in Fig 10 for heavier ions. For estimating the normalized spectra, *φ*_*i*,all_(*E*,*d*) and *φ*_*i*,eva+rec_(*E*), we respectively introduced the following functions:

$$\varphi_{i,\text{all}}\left(E,d\right)=\frac{h_{1,i}\left(d\right)E^{h_{2,i}\left(d\right)}}{\left[1+h_{3,i}\left(d\right)E^{h_{4,i}\left(d\right)}\right]\left[1+h_{5,i}\left(d\right)E^{h_{6,i}\left(d\right)}\right]}\left\{1+\exp\left[-h_{7,i}\left(d\right)\left(\ln\left(E\right)+h_{8,i}\left(d\right)\right)\right]\right\},$$ (6)

and

$$\varphi_{i,\text{eva}+\text{rec}}\left(E\right)=\frac{h_{1,i}E^{h_{2,i}}}{1+h_{3,i}E^{h_{4,i}}}{\left\{1+\exp\left[-h_{7,i}\left(\ln\left(E\right)+h_{8,i}\right)\right]\right\}}^{-1},$$ (7)

where *h*_1,*i*_–*h*_8,*i*_ are free parameters dependent and independent of *d* when calculating *φ*_*i*,all_ and *φ*_*i*,eva+rec_(*E*), respectively. The forms of the equations have little physical meaning; the fraction part represents the power function of *E*, whose power index changes gradually from *h*_2,*i*_ to *h*_2,*i*_-*h*_4,*i*_ or *h*_2,*i*_-*h*_4,*i*_-*h*_6,*i*_ with increasing *E*, and the exponential part represents the correction factor below 1 MeV/n, which was not considered in our previous study.

The numerical value of the *h* parameters were determined from LSq fitting to the evaluated normalized spectra, whose examples are shown as staircases in Figs 9 and 10. The fitting results are also shown in the figures as dashed lines. It is evident from the graphs that Eqs (6) and (7) can reproduce the evaluated normalized spectra very well, even when the flux at 1 MeV/n is not fitted well by Eq (4), for example, in the case of the data of protons and He ions at 0.15 g/cm^2^. This result indicates that the failure of Eq (4) to reproduce the EAS data at lower atmospheric depths, as shown in the right panel of Fig 7, does not result in inaccurate calculation of secondary ion fluxes using Eq (3). Note that the atmospheric-depth dependences of the *h* parameters of protons and He ions were determined by simply interpolating the corresponding evaluated data for discrete altitude levels for which the EAS data were available. By substituting *Φ*_*i*_ and *φ*_*i*_ obtained from Eqs. (4)–(7) into Eq (3), we can analytically estimate the secondary ion fluxes for any global condition.

#### Combining Primary and Secondary Ion Fluxes

In the same manner as our previous study, we employed the following function to calculate the total ion fluxes of particle *i*, *ϕ*_*i*_:

$$\begin{array}{l} \phi_{i}(E,d,r_{\text{c}},W)=\phi_{i,\text{pri}}(E,d,W)\left\{\tanh\left\{o_{1,i}\left[E/E_{\text{s}1,i}\left(r_{\text{c}},d\right)-1\right]\right\}+1\right\}/2\\ \;+\phi_{i,\text{sec}}(E,d,r_{\text{c}},W)\left\{\tanh\left\{o_{2,i}\left[1-E/E_{\text{s}2,i}\left(r_{\text{c}},d\right)\right]\right\}+1\right\}/2, \end{array}$$ (8)

where *ϕ*_*i*,pri_ and *ϕ*_*i*,sec_ are the primary and secondary fluxes calculated using Eqs (2) and (3), respectively, *E*_s1,*i*_ and *E*_s2,*i*_ are the switching energies between the primary and secondary spectra, and *o*_1,*i*_ and *o*_2,*i*_ are free parameters that influence the smoothness of spectrum switching. In general, the switching energy of the primary spectra, *E*_s1_, is equal to that of the secondary spectra, *E*_s2_, and they can be determined from *r*_c_ and *d* as follows:

$$E_{\text{s},i}(r_{\text{c}},d)=o_{3,i}\left[E_{\text{c},i}(r_{\text{c}})-o_{4,i}d\right],$$ (9)

where *o*_3,*i*_ and *o*_4,*i*_ are constant parameters, and *E*_c,*i*_ corresponds to the cut-off energy of the particle *i* at the top of the atmosphere. However, *E*_s,*j*_ occasionally becomes too small, particularly for lower *r*_c_ and larger *d* values, and it is necessary to respectively introduce the minimum values of the switching energy for primary and secondary particles, as follows:

$$\begin{array}{l} E_{\text{s}1,i}(r_{\text{c}},d)=\max\left[o_{5,i},E_{\text{s},i}(r_{\text{c}},d)\right]\\ E_{\text{s}2,i}(r_{\text{c}},d)=\max\left[o_{6,i},E_{\text{s},i}(r_{\text{c}},d)\right] \end{array},$$ (10)

where *o*_5,*i*_ and *o*_6,*i*_ are free parameters that represent the minimum energy. The numerical values of parameters *o*_1,*i*_–*o*_6,*i*_ were determined by LSq fitting with the results of the EAS data, using the primary and secondary fluxes calculated using Eqs (2) and (3), respectively. Note that the values of the *o* parameters were assumed to be the same within the category defined for determining the *b* parameters, as described before.

The fluxes calculated using PARMA3.0, specifically, Eq (8), are also drawn in Figs 1 and 6. It is evident from the graphs that PARMA3.0 can reproduce the corresponding EAS simulation very well, with the exception of the gap regions observed between the primary and secondary fluxes of heavy ions. This discrepancy is ascribed to the fact that Eq (8) was proposed on the basis of the assumption that the energy spectrum of cosmic rays can be expressed by a continuum function, although some EAS data exhibit a sharp cut-off at the switching energy.

### Neutron Fluxes

**Fig 11:**
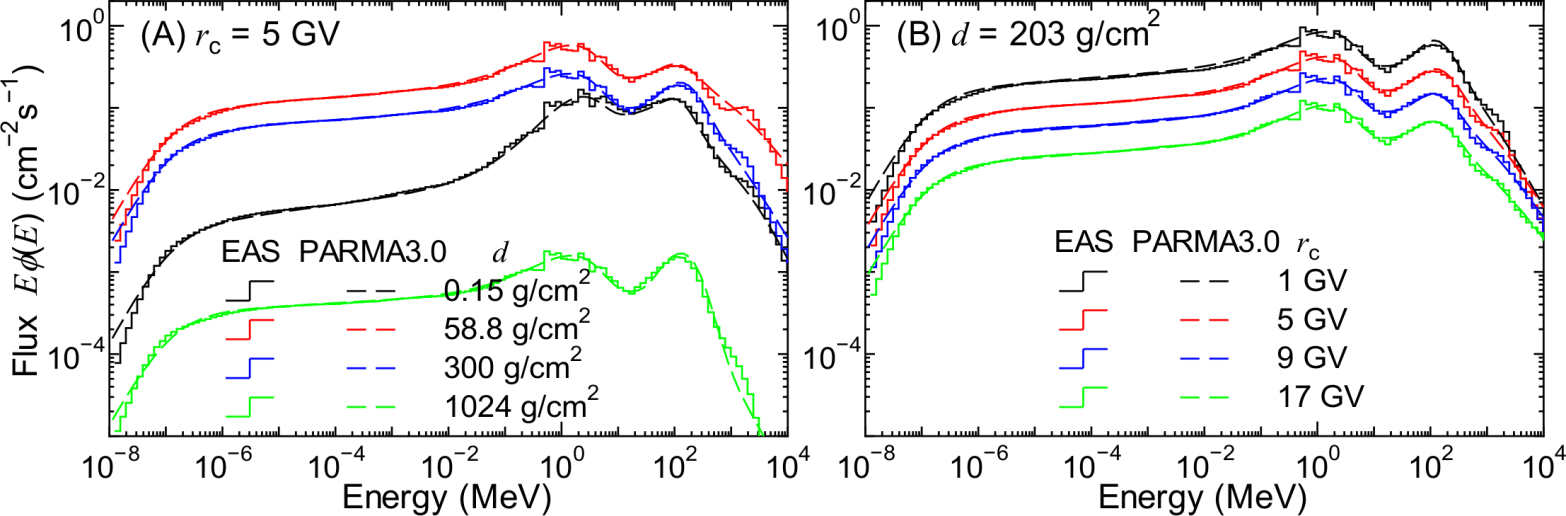
Neutron fluxes obtained by EAS simulation and those calculated using PARMA3.0. The left and right panels show the atmospheric depth and the vertical cut-off rigidity dependences of the fluxes, respectively.

Fig 11 shows the neutron fluxes obtained in the EAS simulation in comparison with the corresponding data calculated using PARMA3.0. In our previous study, we found that the neutron spectra above 20 km significantly vary with altitudes, whereas those below 20 km can be expressed by a simple equation, where the altitude of 20 km corresponds to an atmospheric depth of 56.4 g/cm^2^. Hence, the applicable altitude range of the former versions of PARMA was limited to 20 km. In this study, we first developed a model for estimating neutron fluxes below 20 km in a manner similar to our previous study. Next, we introduced correction factors into the model to extend its applicable altitude range to the top of the atmosphere.

#### Neutron fluxes below 20 km

All neutrons in the atmosphere are secondary particles. Thus, we express the neutron fluxes below 20 km, *ϕ*_n,<20 km_(*E*,*d*,*r*_c_,*W*), using a function similar to Eq (3) as follows:

$$\phi_{\text{n},<20\text{km}}\left(E,d,r_{\text{c}},W\right)=\Phi_{\text{n}}\left(d,r_{\text{c}},W\right)\varphi_{\text{n}}\left(E,d,r_{\text{c}}\right),$$ (11)

where *Φ*_n_(*d*,*r*_c_,*W*) is the normalization flux in cm^−2^s^−1^, and *φ*_n_(*E*,*d*,*r*_c_) is the normalized energy spectrum of neutrons in MeV^−1^, which depends not only on the atmospheric depth, *d* but also on the cut-off rigidity, *r*_c_.

**Fig 12:**
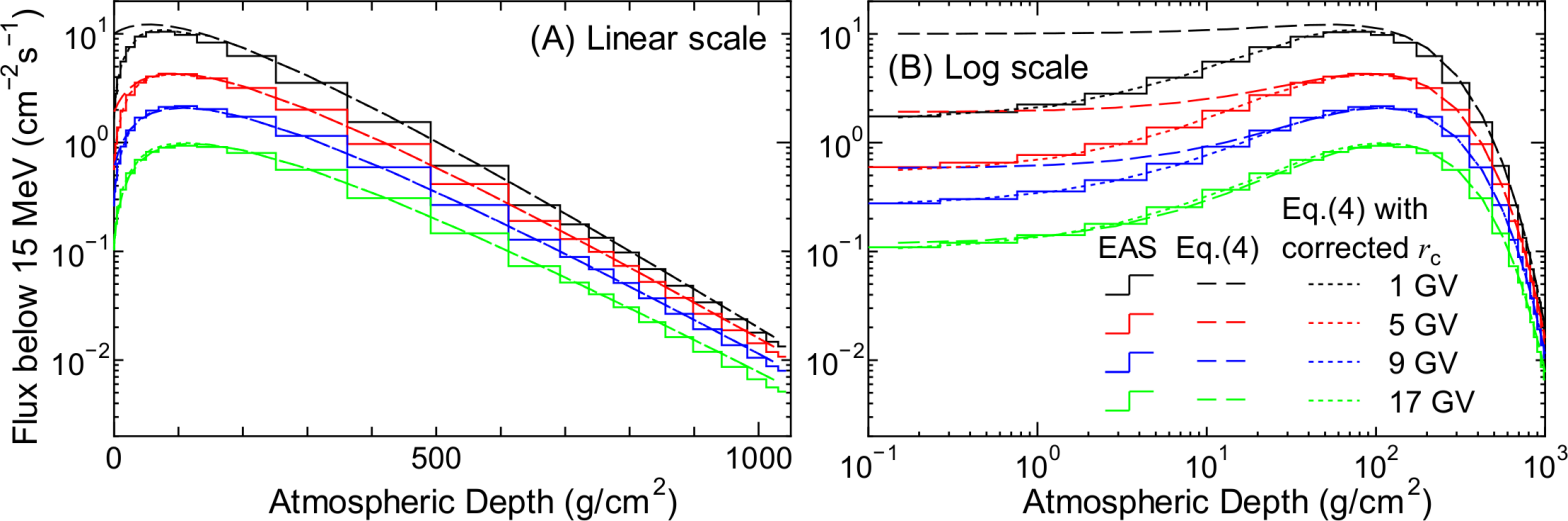
Integrated neutron fluxes below 15 MeV obtained from EAS simulation and corresponding normalization fluxes calculated using Eq (4) before and after correcting *r*_c_. The left and right panels show the same data drawn along different x-axes, in particular, the linear and logarithmic scales of atmospheric depth, respectively.

The normalization flux of neutrons can also be calculated by the combination of Eqs (4) and (5) defined in the previous section; however, in accordance with our previous study, the numerical values of their parameters were fitted to the integrated neutron flux below 15 MeV instead of the flux at 1 MeV obtained from the EAS simulation. The results of the LSq fitting are shown in Fig 12. Panel (A) of the figure clearly indicates that Eq (4) can reproduce the EAS simulation results very well for atmospheric depths greater than50 g/cm^2^. In contrast, the discrepancies between the results of these equations and the EAS simulation for lower atmospheric depths are shown in Panel (B), particularly for smaller cut-off rigidity values. One reason for these discrepancies is that the EAS simulation results at altitudes below 20 km only were employed in the LSq fitting to well reproduce low-altitude data. We compensated for these discrepancies by adjusting the cut-off rigidity when estimating the normalization factors for higher altitudes, as described later in this section.

**Fig 13:**
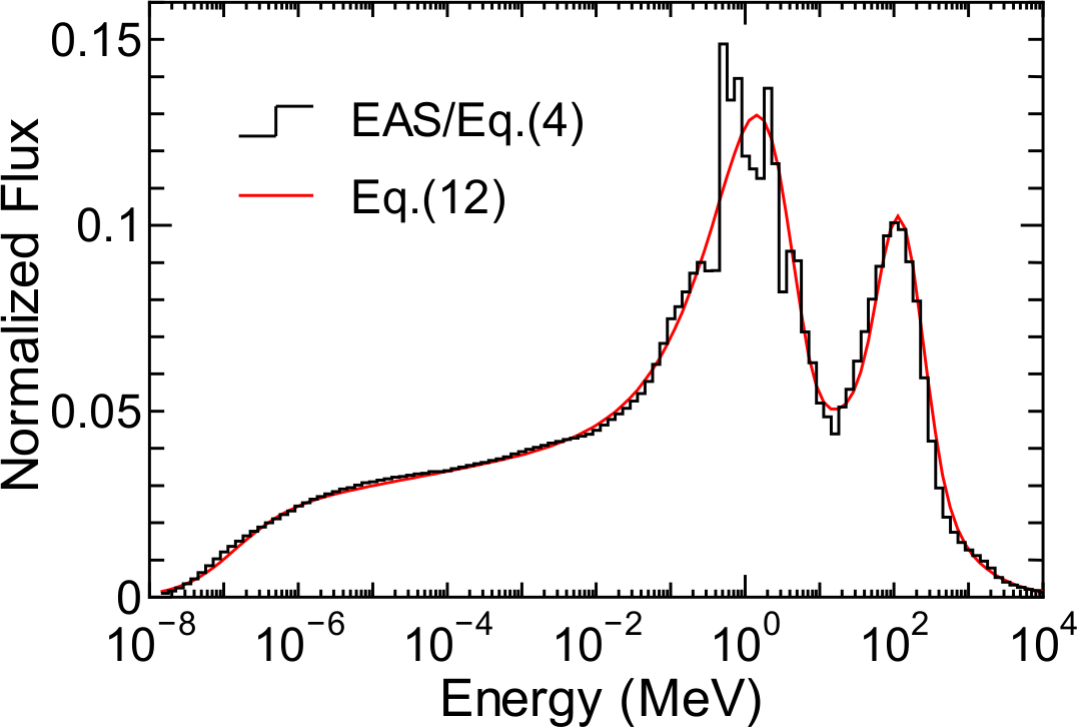
Energy-weighted normalized neutron spectrum, *Eφ*_n_.

Fig 13 shows the energy-weighted normalized neutron spectrum, which is the ratio of energy-weighted neutron fluxes obtained from the EAS simulation divided by *Φ*_n,N_ calculated using Eq (4), averaged over all global conditions except for altitudes higher than 20 km. In the same manner as our previous study, the energy-weighted normalized spectrum of neutrons, *Eφ*_n_, can be expressed as follows:

$$\begin{array}{l} E\varphi_{\text{n}}(E,d,r_{\text{c}})=p_{1}{\left(\frac{E}{p_{2}}\right)}^{p_{3}}\exp\left(\frac{-E}{p_{2}}\right)\\ \;+p_{4}\left(d,r_{\text{c}}\right)\exp\left\{\frac{-{\left[\log_{10}(E)-\log_{10}(p_{5})\right]}^{2}}{2{\left[\log_{10}(p_{6})\right]}^{2}}\right\}\\ \;+p_{7}\log_{10}\left(\frac{E}{p_{8}}\right)\left\{1+\tanh\left[p_{9}\log_{10}\left(\frac{E}{p_{10}}\right)\right]\right\}\left\{1-\tanh\left[p_{11}\log_{10}\left(\frac{E}{p_{12}\left(d,r_{\text{c}}\right)}\right)\right]\right\}, \end{array}$$ (12)

where *p*_1_–*p*_12_ are free parameters. The parameters *p*_4_ and *p*_12_ represent the height of the high-energy peak and the edge of the high-energy tail, respectively. These two parameters are regarded as being dependent on *d* and *r*_c_, while the others are constant under all global conditions. The numerical values of the constant *p* parameters were determined by LSq fitting with the normalized neutron spectrum shown in Fig 13. The *r*_c_ dependence of *p*_4_ and *p*_12_ was also expressed by Eq (5), and the numerical values of the *g* parameters were determined by LSq fitting with the normalized neutron spectrum for each altitude. In contrast, the *d* dependence of *p*_4_ and *p*_12_ was simply expressed by interpolating the evaluated data for discrete altitude levels for which the EAS data were available.

#### Neutron fluxes above 20 km

As mentioned before, the combination of Eqs (4) and (5) fails to reproduce the integrated neutron fluxes below 15 MeV obtained from the EAS simulation for atmospheric depths lower than 50 g/cm^2^. Furthermore, the normalized spectra vary significantly with altitude over 20 km, as shown in Fig 11, and they cannot be expressed using Eq (12). Therefore, we proposed a function to calculate the neutron fluxes at any altitude, *ϕ*_n_, by introducing the concept of best-fit rigidity, *r*_b_, and the spectrum correction factor, *C*_n_, as follows:

$$\phi_{\text{n}}\left(E,d,r_{\text{c}},W\right)=\Phi_{\text{n}}\left(d,r_{\text{b}}\left(r_{\text{c}},d\right),W\right)\varphi_{\text{n}}\left(E,d,r_{\text{c}}\right)C_{\text{n}}\left(E,d,r_{\text{c}},W\right).$$ (13)

The best-fit rigidity represents the *r*_c_ value that gives the closest estimate of the normalization fluxes using Eq (4). This concept was introduced because agreement between the integrated neutron fluxes below 15 MeV obtained from the EAS simulation and those obtained using Eq (4) would be better when a larger cut-off rigidity is substituted in the equation instead of the real value, as shown in Panel (B) of Fig 12. For example, the results calculated using Eq (4) for *r*_c_ = 5 GV agree better with the EAS data for *r*_c_ = 1 GV instead of 5 GV at *d* < 1 g/cm^2^. We evaluated the best-fit rigidity for each global condition by changing the value of *r*_c_ in Eq (4).

**Fig 14:**
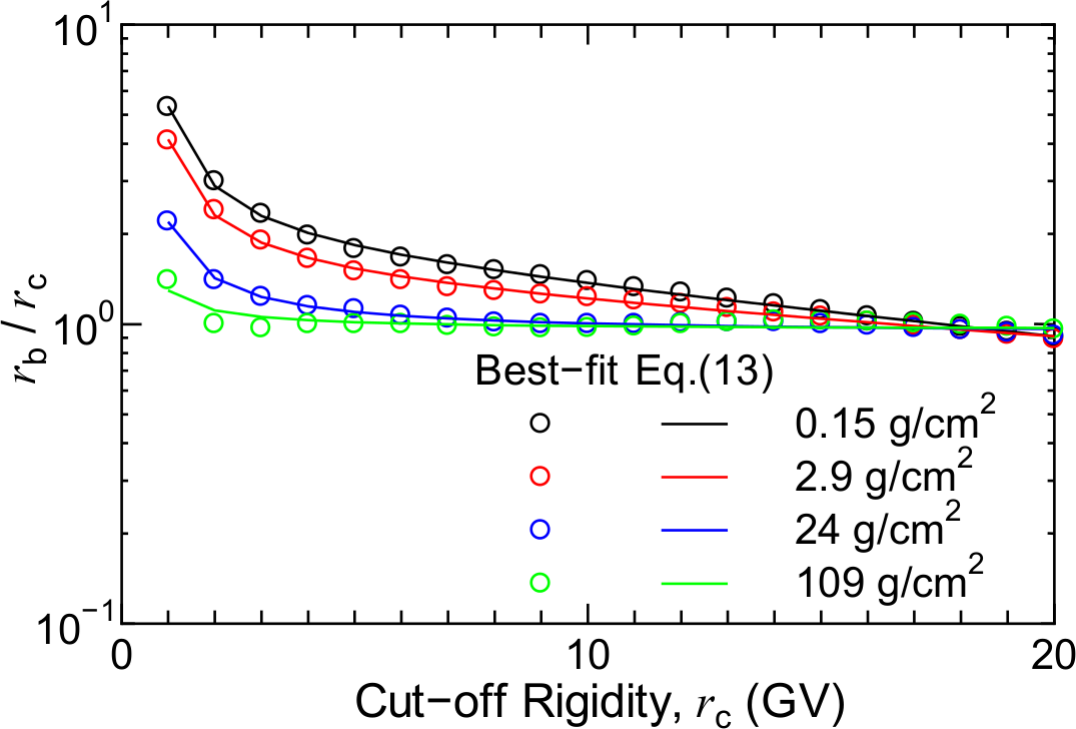
Ratios between best-fit and real cut-off rigidities, *r*_b_/*r*_c_, for solar minimum condition.

Fig 14 shows the ratios between the best-fit and real cut-off rigidities, *r*_b_/*r*_c_, for atmospheric depths below 109 g/cm^2^ under the solar minimum condition. The ratios are very close to 1.0 for *d* = 109 g/cm^2^, indicating that correction of the cut-off rigidity is not necessary for such high atmospheric depths. In contrast, the ratios increase with a decrease in atmospheric depth, particularly for lower cut-off rigidity values. Therefore, we proposed a function to express the relationship between *r*_c_ and r_b_ for the solar minimum and maximum conditions as follows:

$$r_{\text{b}}^{\left(W\mp \right)}\left(r_{\text{c}},d\right)=r_{\text{c}}\times 10^{q_{1}^{\left(W\mp \right)}\left(d\right)+q_{2}^{\left(W\mp \right)}\left(d\right)r_{\text{c}}+q_{3}^{\left(W\mp \right)}\left(d\right)/r_{\text{c}}},$$ (14)

where $q_{1}^{\left(W\mp \right)}$–$q_{3}^{\left(W\mp \right)}$ are free parameters depending on the atmospheric depth *d*. Their numerical values were also determined by LSq fitting with the evaluated $r_{\text{b}}^{\left(W\mp \right)}$, the results of which are shown in Fig 14. The figure clearly indicates the adequacy of the fit. Note that all *q* parameters were set to 0 for *d* > 109 g/cm^2^, i.e., *r*_b_ = *r*_c_ at lower altitudes. The normalization fluxes calculated using Eq (4) by correcting *r*_c_, i.e., substituting the $r_{\text{b}}^{\left(W\mp \right)}$ obtained using Eq (14) in *r*_c_, are also plotted in Fig 13. It is evident from the figure that *r*_c_ correction improves the accuracy of Eq (4) in terms of reproducing the neutron fluxes below 15 MeV for lower *d* and *r*_c_.

**Fig 15:**
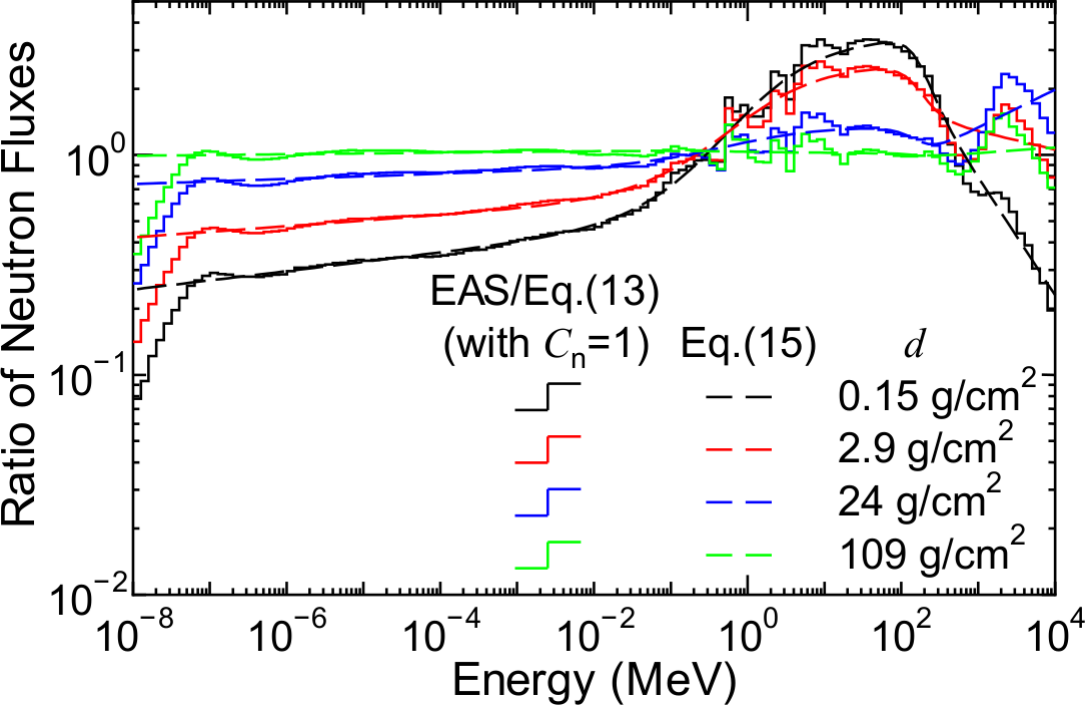
Ratios of neutron fluxes obtained from EAS simulation to those calculated using Eq (13) under assumption of *C*_n_ = 1.0, in comparison with *C*_n_ calculated using Eq (15).

Fig 15 shows the ratios of neutron fluxes obtained from the EAS simulation to those calculated using Eq (13) under the assumption of *C*_n_ = 1.0, which are required to be reproduced by the correction factor *C*_n_. The ratios are very close to 1.0 for *d* = 109 g/cm^2^, indicating that correction of the normalized spectrum is not necessary for such high atmospheric depths. In contrast, the ratios complexly depend on *E*, *d*, *r*_c_, and *W* at lower atmospheric depths. To express the ratios, we proposed a function to calculate *C*_n_ under the solar minimum and maximum conditions as follows:

$$\begin{array}{l} \log_{10}\left[C_{\text{n}}^{\left(W\mp \right)}(E,d,r_{\text{c}})\right]=s_{1}^{\left(W\mp \right)}\left(d,r_{\text{c}}\right)\\ \;+\left[s_{2}^{\left(W\mp \right)}\left(d,r_{\text{c}}\right)\log_{10}\left(E\right)+s_{3}^{\left(W\mp \right)}\left(d,r_{\text{c}}\right)\right]\left\{1-\tanh\left[s_{4}^{\left(W\mp \right)}\left(r_{\text{c}}\right)\log_{10}\left(E/s_{5}^{\left(W\mp \right)}\left(r_{\text{c}}\right)\right)\right]\right\}\\ \;+\left[s_{6}^{\left(W\mp \right)}\left(d,r_{\text{c}}\right)\log_{10}\left(E\right)+s_{7}^{\left(W\mp \right)}\left(d,r_{\text{c}}\right)\right]\left\{1+\tanh\left[s_{8}^{\left(W\mp \right)}\left(r_{\text{c}}\right)\log_{10}\left(E/s_{9}^{\left(W\mp \right)}\left(r_{\text{c}}\right)\right)\right]\right\}, \end{array}$$ (15)

where $s_{1}^{\left(W\mp \right)}$–$s_{9}^{\left(W\mp \right)}$ are free parameters depending on *r*_c_ and/or *d*, and their dependences are expressed by Eq (5) and the interpolation method, respectively, in the same manner as for the other free parameters used in PARMA3.0. The numerical values of the parameters used for calculating $s_{1}^{\left(W\mp \right)}$–$s_{9}^{\left(W\mp \right)}$ were determined by LSq fitting with the flux ratios, the examples of which are shown in Fig 15, and the results of the LSq fitting are also shown in the figure. The agreement between the flux ratios and *C*_n_ calculated using Eq (15) is quite satisfactory, although slight discrepancies are observed for neutron energies above 1 GeV. To calculate *Φ*_n,N_ and *C*_n_ for arbitrary solar conditions, we used Eqs (8)–(10) in [21] as well.

The neutron fluxes calculated using PARMA3.0, specifically, Eq (13), are also drawn in Figs 1 and 11. It is evident from the graphs that PARMA3.0 can reproduce the corresponding EAS simulation very well, even at higher altitudes, where the former version of PARMA cannot be applied. It should be mentioned that Eq (13) enables calculation of the neutron fluxes only under the ideal condition, and the conversion function for considering the local geometry effect proposed in our previous study must be used for estimating the neutron fluxes near ground or in aircraft. The neutron fluxes shown by dashed lines in Fig 2 were calculated by PARMA3.0 considering the local geometry effect. It can be observed from the graphs that PARMA3.0 is substantially superior to the EAS simulation in reproducing the experimental data because it considers the local geometry effect.

### Electron, Positron, and Photon Fluxes

**Fig 16:**
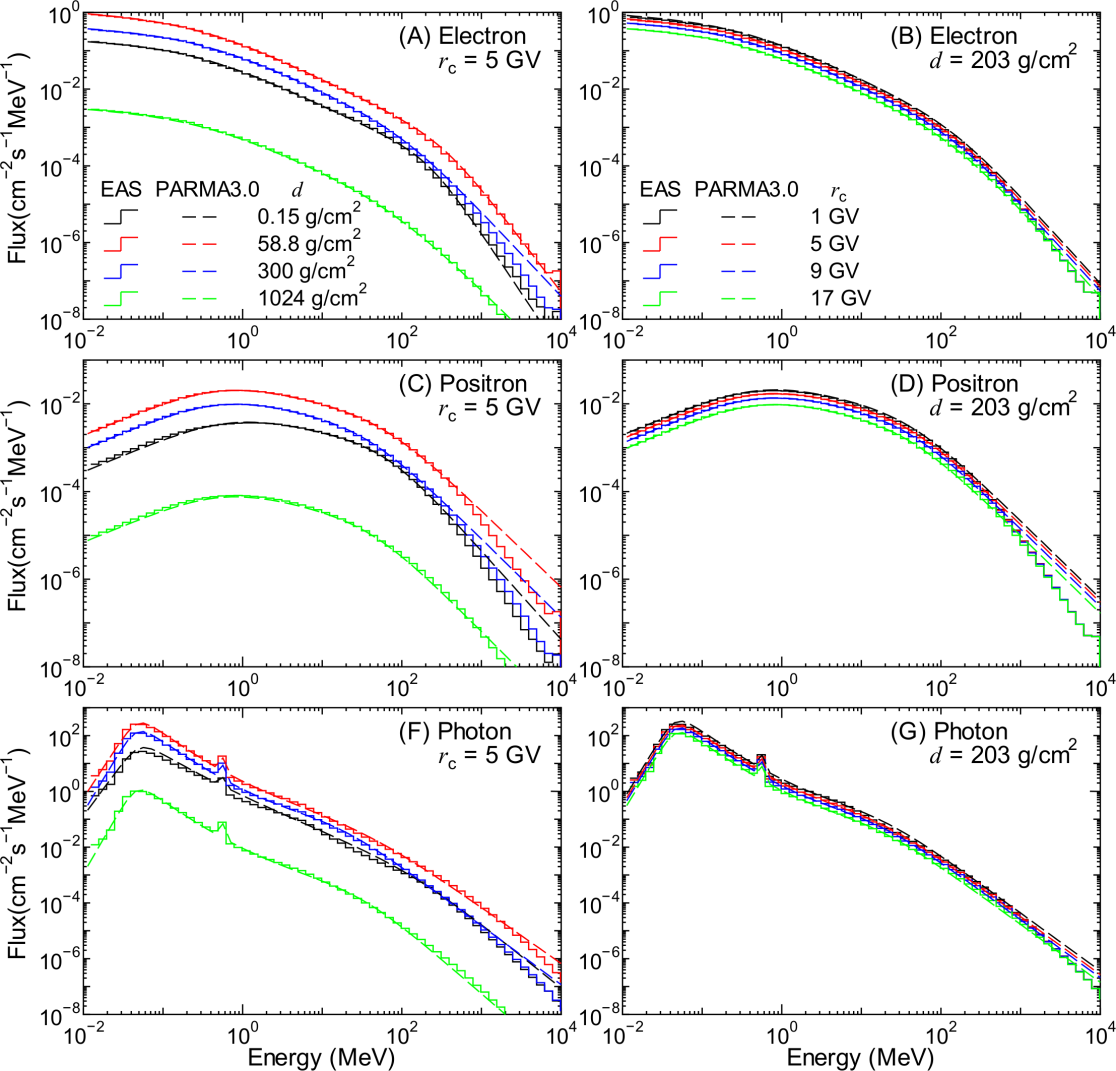
Electron, positron, and photon fluxes obtained from EAS simulation and PARMA3.0. The left and right panels indicate the atmospheric depth and the vertical cut-off rigidity dependences of the fluxes, respectively.

Fig 16 shows the electron, positron, and photon fluxes obtained by the EAS simulation along with the corresponding data calculated using PARMA3.0. The left and right panels show the atmospheric depth and the vertical cut-off rigidity dependences of the fluxes, respectively. The small peaks observed in the photon fluxes at approximately 0.5 MeV are attributed to the production of annihilation γ-rays.

Electrons, positrons, and photons exist in space, but they were not considered as the primary particles in our EAS simulation. Thus, their fluxes in the atmosphere, *ϕ*_*i*_(*E*,*d*,*r*_c_,*W*), can be expressed by the same functions used to calculate the secondary ion fluxes as follows:

$$\phi_{i}\left(E,d,r_{\text{c}},W\right)=\Phi_{i}\left(d,r_{\text{c}},W\right)\varphi_{i}\left(E,d\right)\;\text{for}\;i=e^{-},\;e^{+},\;\text{and}\;\gamma$$ (16)

where *Φ*_*i*_(*d*,*r*_c_,*W*) is the normalization flux in cm^−2^s^−1^ and *φ*_*i*_(*E*,*d*) is the normalized energy spectrum in MeV^−1^. The numerical values of *Φ*_*i*_ were also fitted to the flux of particle *i* at 1 MeV using the combination of Eqs (4) and (5), similar to for ions, but the concept of best-fit rigidity was introduced in the calculation for high-altitude data in the same manner as the neutron case.

**Fig 17:**
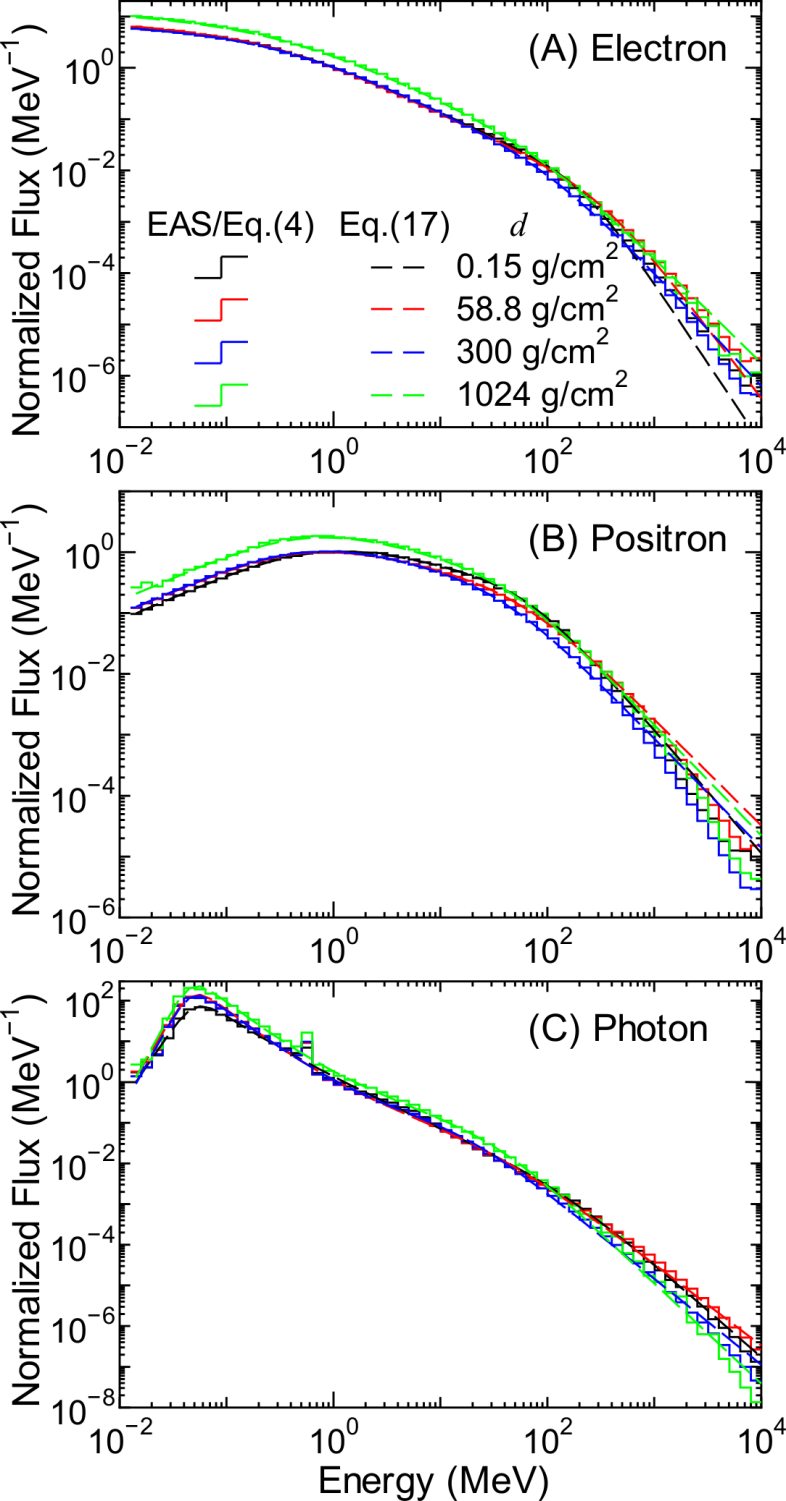
Normalized spectra of (A) electrons, (B) positrons, and (C) photons for each atmospheric depth.

Fig 17 shows the normalized energy spectra for electrons, positrons, and photons, which are the ratios of the fluxes obtained from the EAS simulation to *Φ*_*i*_ calculated using Eq (4). We expressed the normalized energy spectra for electrons and positrons, *φ*_(e∓)_ as follows:

$$\varphi_{\left(\text{e}\mp \right)}\left(E,d\right)=\frac{h_{1,(\text{e}\mp )}\left(d\right)E^{h_{2,(\text{e}\mp )}\left(d\right)}}{\left[1+h_{3,(\text{e}\mp )}\left(d\right)E^{h_{4,(\text{e}\mp )}\left(d\right)}\right]\left[1+h_{5,(\text{e}\mp )}\left(d\right)E^{h_{6,(\text{e}\mp )}\left(d\right)}\right]},$$ (17)

where *h*_1,(e∓)_–*h*_8,(e∓)_ are free parameters depending on *d*. The form of this equation is similar to that of Eq (6), but the low-energy correction factor is not included. In contrast, the normalized energy spectrum for photons, *φ*_γ_, is expressed as follows:

$$\varphi_{\gamma }\left(E,d\right)=\frac{h_{1,\gamma }\left(d\right)E^{h_{2,\gamma }\left(d\right)}\left[1+h_{3,\gamma }\left(d\right)E^{h_{4,\gamma }\left(d\right)}\right]}{\left[1+h_{5,\gamma }\left(d\right)E^{h_{6,\gamma }\left(d\right)}\right]}\left\{1+\exp\left[-h_{7,\gamma }\left(d\right)\left(\ln\left(E\right)+h_{8,\gamma }\left(d\right)\right)\right]\right\}+h_{9,\gamma }\left(d\right)\delta \left(E_{\text{a}}\right),$$ (18)

where *δ* is Dirac’s delta function and *E*_a_ is the energy of the annihilation γ-rays, which is equal to the rest mass of the electron, i.e., 0.511 MeV. The numerical values of the *h* parameters were determined by LSq fitting with the evaluated normalized spectra, the examples of which are shown as staircases in Fig 17. The fitting results are also shown in the figures by dashed lines. It is evident from the graphs that Eqs (17) and (18) can reproduce the evaluated normalized spectra very well, with the exception of very high energies over 1 GeV. Note that the fitting results shown in the figure are the mean values for certain energy bins; otherwise, the photon spectra at *E* = 0.511 MeV have infinite value.

The fluxes calculated using PARMA3.0, specifically, Eq (16), are also shown in Figs 1 and 16. The agreement between the results obtained from the EAS simulation and PARMA3.0 is quite satisfactory, although slight disagreements are observed for energies over 1 GeV. However, fluxes of such high-energy electrons, positrons, and photons are generally very small, and the effect of these discrepancies in the practical use of PARMA3.0 can be considered negligible.

### Muon Fluxes

**Fig 18:**
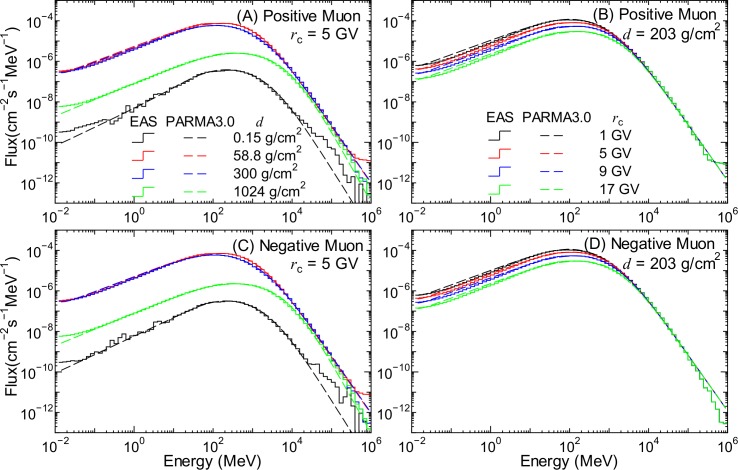
Positive and negative muon fluxes obtained from EAS simulation and PARMA3.0. The left and right panels indicate the atmospheric depth and the vertical cut-off rigidity dependences of the fluxes, respectively.

Fig 18 shows the positive and negative muon fluxes obtained by the EAS simulation together with the corresponding data calculated using PARMA3.0. Our previous study suggested that the muon fluxes over 10 GeV are almost independent of *r*_c_ and *W* because such high-energy muons are produced by higher-energy primary cosmic rays, whose fluxes are not influenced by those global conditions.

Similar to other secondary particles, we proposed a function for estimating the muon fluxes, *ϕ*_(μ∓)_(*E*,*d*,*r*_c_,*W*) as follows:

$$\phi_{\left(\mu \mp \right)}\left(E,d,r_{\text{c}},W\right)=\Phi_{\left(\mu \mp \right)}\left(d\right)\varphi_{\left(\mu \mp \right)}\left(E,d,r_{\text{c}},W\right),$$ (19)

where *Φ*_(μ∓)_(*d*) is the normalization flux in cm^−2^s^−1^ and *φ*_(μ∓)_(*E*,*d*,*r*_c_,*W*) is the normalized energy spectrum in MeV^−1^. In the estimation of muon fluxes, the normalization flux is related to their high-energy fluxes, which are almost independent of *r*_c_ and *W*, as shown in Fig 18. Thus, *Φ*_(μ∓)_ is regarded as a free parameter depending only on *d* in this study.

Based on Lipari’s model [54], we expressed the normalized energy spectra, *φ*_(μ∓)_ for the solar minimum and maximum conditions as follows:

$$\begin{array}{l} \varphi_{\left(\mu \mp \right)}^{\left(W\mp \right)}\left(E,d,r_{\text{c}}\right)={\left[E+\frac{t_{1,(\mu \mp )}^{\left(W\mp \right)}\left(d,r_{\text{c}}\right)+t_{2,(\mu \mp )}\left(d\right)\log_{10}\left(E\right)}{\beta^{t_{3,(\mu \mp )}^{\left(W\mp \right)}\left(d,r_{\text{c}}\right)}}\right]}^{t_{4,(\mu \mp )}\left(d\right)}\\ \;\times \left\{1+\exp\left[-t_{5,(\mu \mp )}^{\left(W\mp \right)}\left(d,r_{\text{c}}\right)\left(\ln\left(E\right)+t_{6,(\mu \mp )}^{\left(W\mp \right)}\left(d,r_{\text{c}}\right)\right)\right]\right\}, \end{array}$$ (20)

where *β* is the speed of muons relative to light and $t_{1,(\mu \mp )}^{\left(W\mp \right)}$–$t_{6,(\mu \mp )}^{\left(W\mp \right)}$ are free parameters depending on *d* as well as *r*_c_ in some cases. The detailed derivation of this equation from Lipari’s model was given in our previous paper. The correction factor for low energies, represented by the second line of the equation, was introduced in this study for extending the applicable energy down to 10 keV. The dependences of the *t* parameters on *r*_c_ and *d* were expressed by Eq (5) and the interpolation method in the same manner as the other free parameters used in PARMA3.0. The numerical values of *Φ*_(μ∓)_ as well as the parameters used for calculating $t_{1,(\mu \mp )}^{\left(W\mp \right)}$–$t_{6,(\mu \mp )}^{\left(W\mp \right)}$ were determined by LSq fitting with the muon fluxes obtained from the EAS simulation. To calculate *ϕ*_(μ∓)_ for arbitrary solar conditions, we also employed Eqs (8)–(10) given in Ref. [21].

The muon fluxes calculated by PARMA3.0, specifically, Eq (19), are also shown in Figs 1 and 18. It is evident from the graphs that PARMA3.0 can reproduce the corresponding EAS simulation very well, with the exception of energies over 100 GeV. In general, PARMA3.0 slightly overestimates the EAS simulation results for such high energies except for *d* = 0.15 g/cm^2^. This is because such high-energy muons are generally produced by nuclear reactions induced by primary cosmic rays of energies over 1 TeV/n, which were not considered in our EAS simulation. According to theory, high-energy muon fluxes can be expressed simply using a power function of muon energy, and hence, the PARMA3.0 results are more reliable than the corresponding EAS data in such high-energy regions.

## Verification and Validation of PARMA3.0

### Verification of PARMA3.0

To quantitatively verify the accuracy of PARMA3.0 in reproducing the EAS data, we calculated the coefficients of determination, *R* ^2^, for each particle and global condition using the following equation:

$$R^{2}=1-\frac{\sum_{k}{\left[E_{k}\phi_{i,E_{k},\text{EAS}}-E_{k}\phi_{i}\left(E_{k}\right)\right]}^{2}}{\sum_{k}{\left[E_{k}\phi_{i,E_{k},\text{EAS}}-E_{k}\bar{\phi_{i,E_{k},\text{EAS}}}\right]}^{2}},$$ (21)

where $\phi_{i,E_{k},\text{EAS}}$ is the EAS data of the flux of particle *i* for *k*-th energy bin, whose central energy is *E*_k_, $\bar{\phi_{i,E_{k},\text{EAS}}}$ is the mean value of $\phi_{i,E_{k},\text{EAS}}$, and *ϕ*_*i*_ (*E*_*k*_) is the corresponding data calculated using PARMA3.0. The reason for selecting *Eϕ* instead of *ϕ* in the calculation of *R* ^2^ was that *Eϕ* is roughly proportional to the integrated flux within the energy bin in the case of the logarithmic energy mesh, and the reproduction of the integrated flux is more important than that of the differential flux in the practical use of the model.

Table 1 summarizes the calculated *R* ^2^ averaged over all global conditions for protons, He ions, neutrons, electrons, positrons, photons, and positive and negative muons, as well as their standard deviations. The calculated *R* ^2^ averaged over all heavy ions with *Z* ≥ 3 is also given in the table, but *R* ^2^ for altitudes below 20 km was excluded from the mean-value calculation because of the large statistical uncertainties in the EAS data under such conditions.

**Table 1:** Coefficients of determination, *R* ^2^, calculated using Eq (21).

	*Mean	**S.D.
Proton	0.972	0.0317
He ion	0.891	0.296
Neutron	0.973	0.0055
Electron	0.993	0.0123
Positron	0.987	0.0223
Photon	0.969	0.0326
Positive Muon	0.990	0.0188
Negative Muon	0.988	0.0333
Heavy ions with Z ≥ 3	0.846	0.262

*Calculated *R* ^2^ averaged over all global conditions except for heavy ions with Z ≥ 3, where the data for altitudes below 20 km were excluded from the mean-value calculation.

**Standard deviation of the mean value.

The calculated *R* ^2^ values are very close to 1.0, except for those of He and heavy ions. The smaller *R* ^2^ for those ion data are predominantly due to failure in reproducing the ion fluxes in the gap region between the primary and secondary particles, as shown in Fig 6. However, such discrepancies are observed only in the data for some global conditions, and approximately80% and 50% of the *R* ^2^ values are greater than 0.9 even for the He and heavy-ion data, respectively. Based on these considerations, we concluded that PARMA3.0 allows for instantaneous estimation of the cosmic ray fluxes under most global conditions with accuracy equivalent to that of the EAS simulation.

### Validation of PARMA3.0

#### Indexes for expressing global conditions

The indexes for expressing the global conditions employed in PARMA3.0 are atmospheric depth *d*, vertical cut-off rigidity *r*_c_, and solar index *W*. To validate PARMA3.0 for various purposes such as route-dose calculation, it is necessary to deduce these parameters using more popular indexes, for example, altitude, longitude, latitude, and date of interest. Ignoring the temporal variation of air pressure, an altitude can be easily converted to the corresponding atmospheric depth using US Standard Atmosphere 1976. The vertical cut-off rigidity at a certain latitude and longitude can be estimated with the worldwide cut-off rigidity map, which was developed using MAGNETOCOSMICS [55]. This map is also included in EXPACS.

As mentioned before, the value of *W* can be determined from cosmic ray measurements and neutron monitor count rates in the Matthiä model. In their study [35], the time variation of *W* was determined from the measurements of Advanced Composition Explorer (ACE) spacecraft [56]; the results were then fitted using a linear function of the count rates of the Oulu neutron monitor [57], *C*_oulu_ as follows:

$$W_{\text{oulu}}=u_{1,\text{oulu}}C_{\text{oulu}}+u_{2,\text{oulu}},$$ (22)

where *W*_oulu_ is the *W* index deduced from the count rates of the Oulu neutron monitor, and *u*_1_ and *u*_2_ are free parameters whose numerical values were evaluated to be −0.093 and 638.7, respectively; supplying *C*_oulu_ in counts/min. In the same manner, we evaluated the *u* parameters of the McMurdo, Newark, South Pole, Thule [58], and Climax [59] neutron monitors as well as the Oulu station. Then, the daily variation of *W* since 1951 was estimated by calculating the mean value of *W* obtained from each station. The evaluated *u* parameters are listed in Table 2. Note that our evaluated *u* parameters for the Oulu station are slightly different from the corresponding data of Matthiä et al. because we excluded the neutron monitor data during ground-level enhancements (GLE) in LSq fitting. It should be mentioned that the *W* values during GLE events cannot be precisely estimated using this method.

**Table 2:** Evaluated *u* parameters used in Eq (22) in the case of supplying the count rate of each neutron monitor in count/min.

Station Name	*u*_1_	*u*_2_
McMurdo	−0.0540	571
Newark	−0.177	659
South Pole	−0.0410	486
Thule	−0.133	629
Climax	−0.121	549
Oulu	−0.0931	639

**Fig 19:**
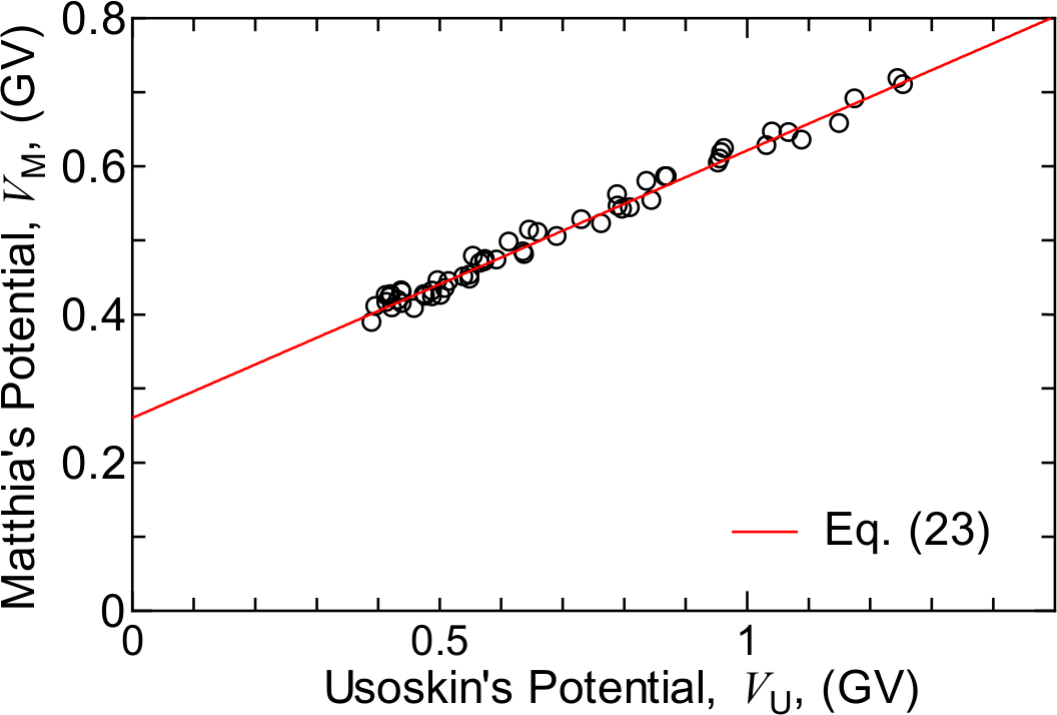
Relation between annual solar modulation potentials after 1951 evaluated by Usoskin et al. [60], *V*_U_, and the corresponding data used in Matthiä model. Matthiä’s potential, *V*_M_, was calculated from our evaluated *W* values using the relationship: *V*_M_ = 0.37 + 0.0003 *W* ^1.45^.

The *W* values before 1951 were determined from reconstructed cosmic ray intensity, as proposed by Usoskin et al. [60]. In their study, the annual solar modulation potentials of the last 400 years were evaluated from the corresponding sunspot numbers, with the exception of the Maunder Minimum period. Fig 19 shows the relationship between their evaluated annual solar modulation potentials after 1951, *V*_U_, and the corresponding data used in the Matthiä model, *V*_M_, which were calculated from our evaluated *W* values using the relationship *V*_M_ = 0.37 + 0.0003 *W* ^1.45^. The figure clearly indicates that the relationship between these two potentials is linear. Based on LSq fitting of the plotted data, we concluded that the relationship between the solar modulation potentials used in the two models can be expressed as follows:

$$V_{\text{M}}=0.260+0.361V_{\text{U}}.$$ (23)

The annual *W* values of the last 400 years except for the Maunder Minimum period can be deduced from the *V*_M_ values calculated using this equation.

### Radiation Dose

**Fig 20:**
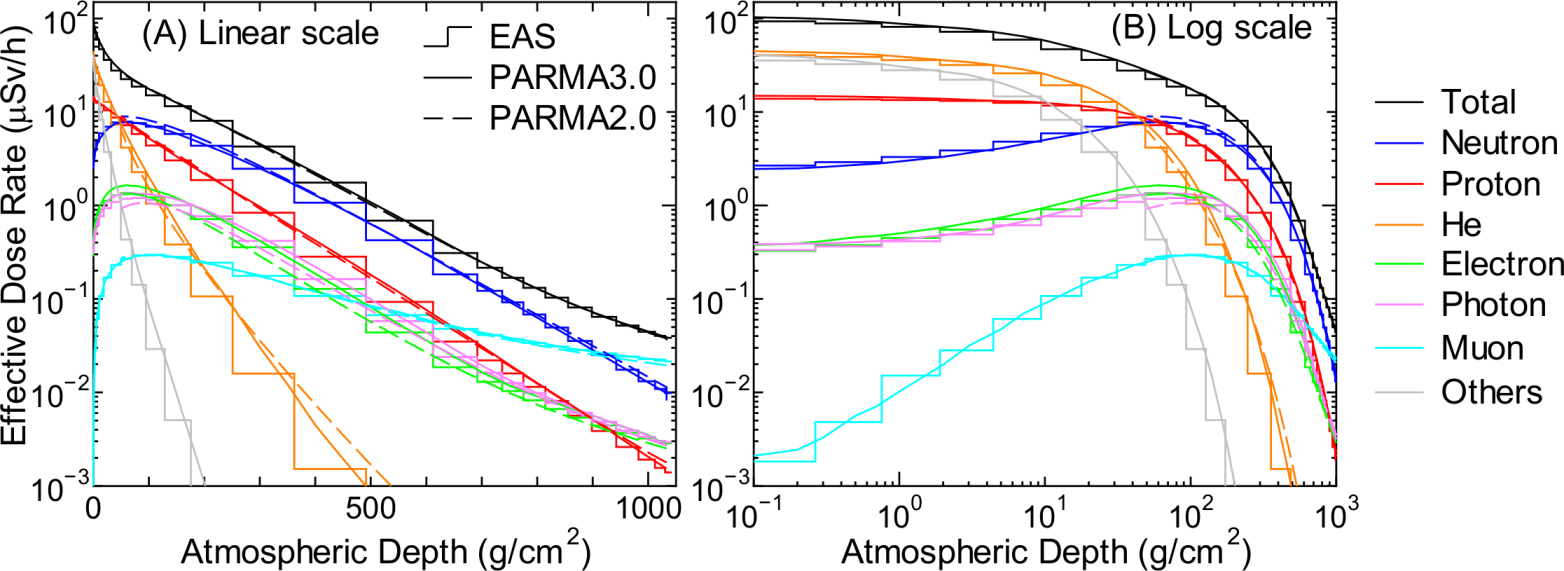
Atmospheric depth dependences of effective dose rates obtained from EAS simulation and calculated using PARMA2.0/3.0 at *r*_c_ = 0 GV for solar minimum condition. The left and right panels show the same data drawn along different x-axes, in particular, the linear and logarithmic scales of atmospheric depth, respectively.

**Fig 21:**
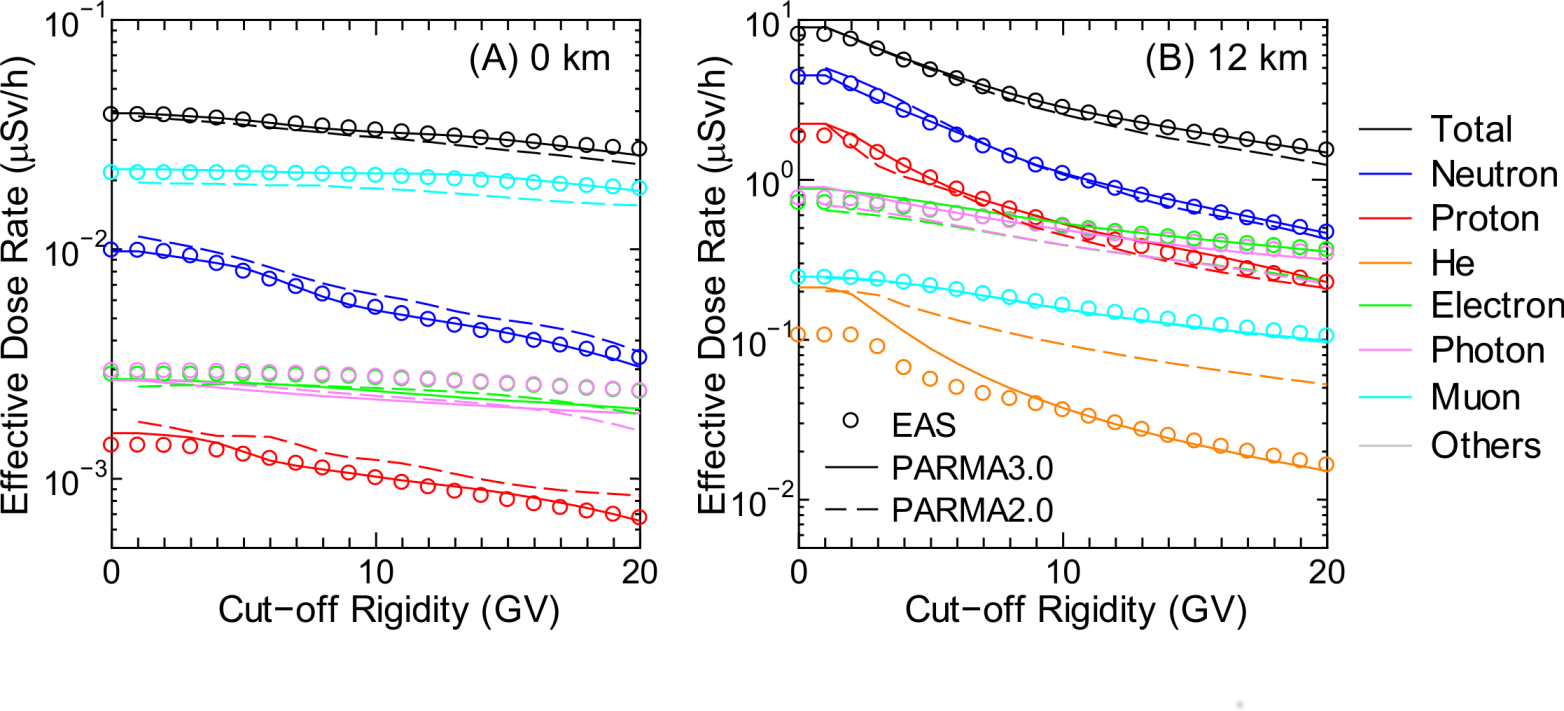
Cut-off rigidity dependences of effective dose rates obtained from EAS simulation and calculated using PARMA2.0/3.0 for solar minimum condition. The left and right panels show the data corresponding to altitudes of 0 and 12 km, respectively.

**Fig 22:**
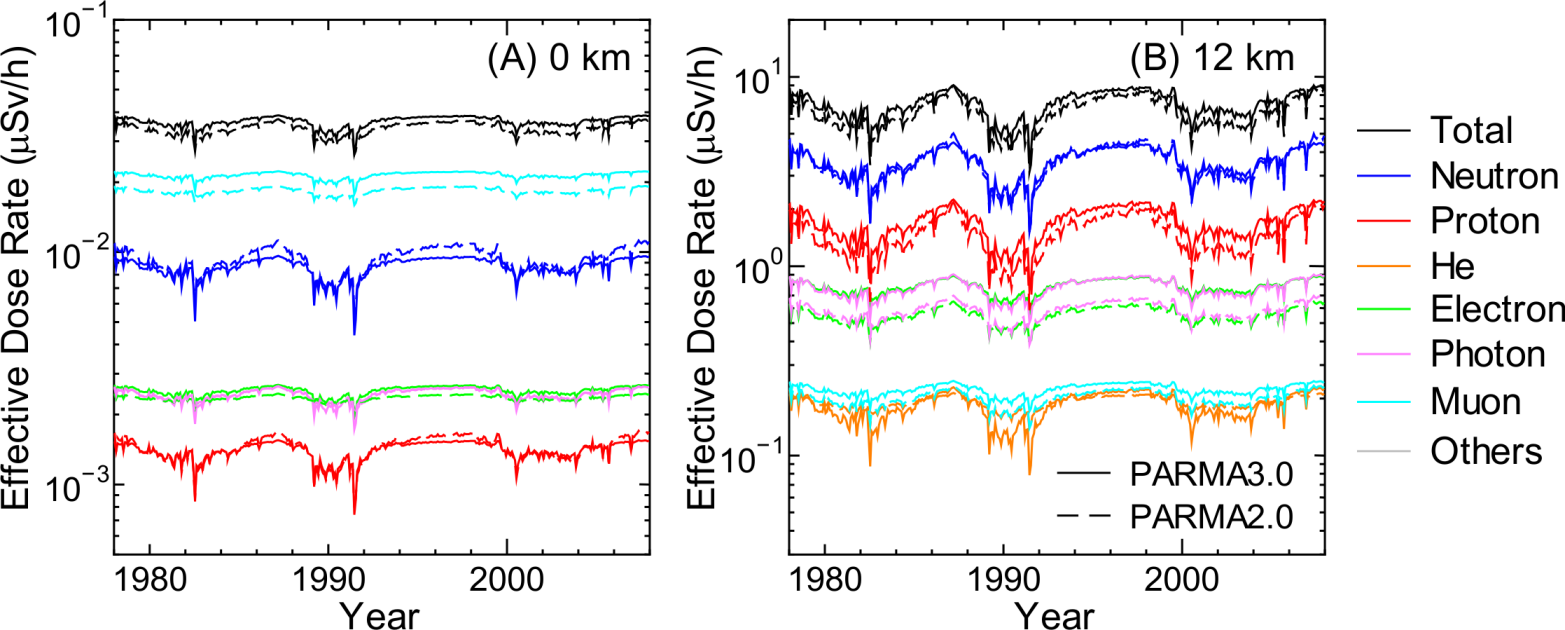
Time dependences of effective dose rates calculated by PARMA2.0/3.0 at *r*_c_ = 0 GV. The left and right panels show the data corresponding to altitudes of 0 and 12 km, respectively.

Estimation of radiation doses to aircrews as well as public is one of the most important applications of PARMA. Figs 20–22 show the effective dose rates calculated using PARMA3.0 for various global conditions in comparison with the corresponding data obtained from the EAS simulation. The results obtained from the previous version of PARMA, PARMA2.0 [21], are also shown in the figures. The fluence to effective dose conversion coefficients for the isotropic irradiation geometry specified in the International Committee on Radiological Protection (ICRP) Publications 116 and 123 [61,62] were employed for dose estimation. Note that the radiation weighting factors defined in ICRP Publication 103 [63] were adopted for expressing the radiation qualities of all particles including heavy ions, although these factors are known to be inadequate to be applied in space dosimetry, as discussed in ICRP 123.

As expected from the discussion of the above *R* ^2^ analysis, excellent agreements were observed between the dose rates obtained using PARMA3.0 and the EAS simulation data, with the exception of He-ion data, where PARMA3.0 overestimated the EAS data under some conditions, for example, lower *r*_c_ at 12 km, as shown in Panel (B) of Fig 21. This exception is attributable to the failure to reproduce the ion fluxes in the gap region between the primary and secondary particles. The figures also suggest that the two versions of PARMA give very similar results, with the exception of He-ion, electron, positron, and photon data. The agreements between the total doses obtained using the two models are less than 5% in most cases. The He-ion doses generally decrease in the new version because the nuclear reaction model for high-energy ions was improved in PHITS2.73. In contrast, the relationships are reversed for electron, positron, and photon doses because the contributions of particles below 1 MeV, which are considered only in PARMA3.0, are non-negligible for those particles.

For more direct validation of PARMA3.0 from the viewpoint of dose estimation, we compared the ambient dose equivalents, H*(10), measured on ground and in aircraft [42,43,64–66] with the corresponding data calculated using PARMA3.0. The influence of ground or aircraft was considered in the calculation. The results of the comparison are summarized in Table 3. It is evident from the table that PARMA3.0 can reproduce the experimental data fairly well over wide ranges of altitude, cut-off rigidity values, and solar modulation. More comprehensive validation will be feasible when the model is implemented in route-dose calculation systems such as JISCARD.

**Table 3:** H*(10) measured on ground and in aircraft [42,43,64–66] in comparison to corresponding data calculated using PARMA3.0.

Reference	Particle	Instrument	*d* (g/cm^2^)	*r*_c_ (GV)	*W*	Exp.(μSv/h)	Cal.(μSv/h)
Goldhagen et al. [39]	Neutron	Bonner Ball	53.5	11.4	30.9	1.1	1.3
			56	0.8	30.9	8.5	8.4
			101	0.7	30.9	7.8	7.9
			201	4.5	30.9	2.7	2.9
			1030	2.7	30.9	0.0093	0.010
Nakamura et al. [40]	Neutron	Bonner Ball	221	12.0	63.9	0.808	0.82
			559	12.0	63.9	0.158	0.14
			1026	10.0	102.5	0.00647	0.0060
Kowatari et al. [61]	Neutron	Bonner Ball	1025	8.6	106.2	0.0082	0.0060
			965	7.1	106.2	0.0076	0.0073
			923	7.7	106.2	0.010	0.0093
			856	7.1	106.2	0.016	0.017
			785	13.3	122.5	0.0054	0.0046
			923	13.5	122.5	0.0053	0.0045
			856	13.4	122.5	0.0077	0.0076
			785	13.4	122.5	0.011	0.012
Latocha et al. [62]	All	*TEPC (ARCS & IRSN)	192	1.8	101.8	7.4 & 6.5	6.4
			280	1.8	101.8	4.6 & 3.8	3.9
			192	6.4	105.1	4.2 & 4.2	4.2
			280	6.4	105.1	2.9 & 2.8	2.7
Wissmann et al. [63]	All	**TEPC (πDOS & HAWK)	192	1.2	15.1	9.5 & 8.9	9.4
			280	1.2	15.1	5.0 & 4.9	5.4
			192	3.9	15.9	7.1 & 6.3	6.4
			280	3.9	15.9	4.2 & 4.3	4.0

*Two TEPCs operated by Austrian Research Center Seibersdorf (ARCS) and Institute for Radiological Protection and Nuclear Safety (IRSN) were mounted on an aircraft.

**Two TEPCs called πDOS and HAWK were mounted on an aircraft.

#### Cosmic Ray-Induced Ionization

Estimating the cosmic ray-induced ionization rate is very important in the theory of cosmolimatology. Therefore, we calculated cosmic ray-induced ionization rates under various global conditions using PARMA3.0 and compared the results with the measured data [67,68]. Except for electrons and positrons, unrestricted collision stopping power in dry air was employed for converting particle flux to ionization rate because the production of knock-out electron, so-called δ-ray, was not explicitly considered in our EAS simulation. In the cases of electrons and positrons, restricted collision stopping power below 10 keV was adopted for the conversion to avoid double counting of the contributions of higher-energy electrons. The mean energy of ion pair creation was assumed to be 35 eV, which is the value employed by Usoskin et al. [69].

**Fig 23:**
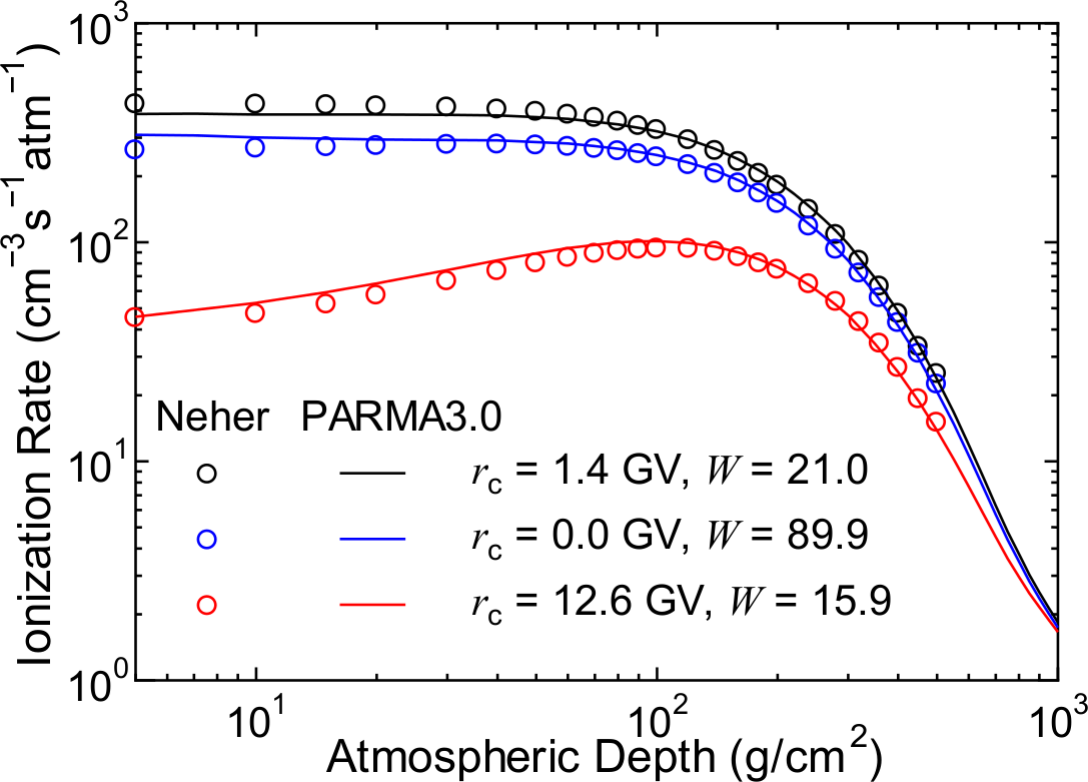
Cosmic ray-induced ionization rates for various global conditions measured by Neher [67,68] and calculated using PARMA3.0.

The results of the comparison are shown in Fig 23. It is clear that PARMA3.0 can reproduce very well the cosmic ray-induced ionization rates measured under various global conditions. Unlike other models for calculating cosmic ray-induced ionization, PARMA3.0 can determine the charge and energy of the particle contributing to the ionization. This feature might be advantageous if the nucleation rate depends not only on the total number of ion pairs created but also on their spatial distribution because highly ionizing particles densely produce ion pairs only around their trajectories.

#### Count Rate of Neutron Monitor

Numerous neutron monitors are continuously operated to measure long-term variations in cosmic ray intensities and to detect ground-level enhancements induced by large solar proton events, and it is worthwhile to reproduce the count rates of each monitor by simulation. Therefore, we calculated the count rates of the neutron monitors in Thule, Newark, and South Pole, and compared them with the observed values [58]. The detection efficiencies of the neutron monitors calculated by PHITS [41] were employed for converting particle fluxes to count rates. It should be mentioned that the count rates significantly depend on the water density in ground, and they were assumed to be 20% for Thule and Newark, and 100% for the South Pole because it is on ice.

**Fig 24:**
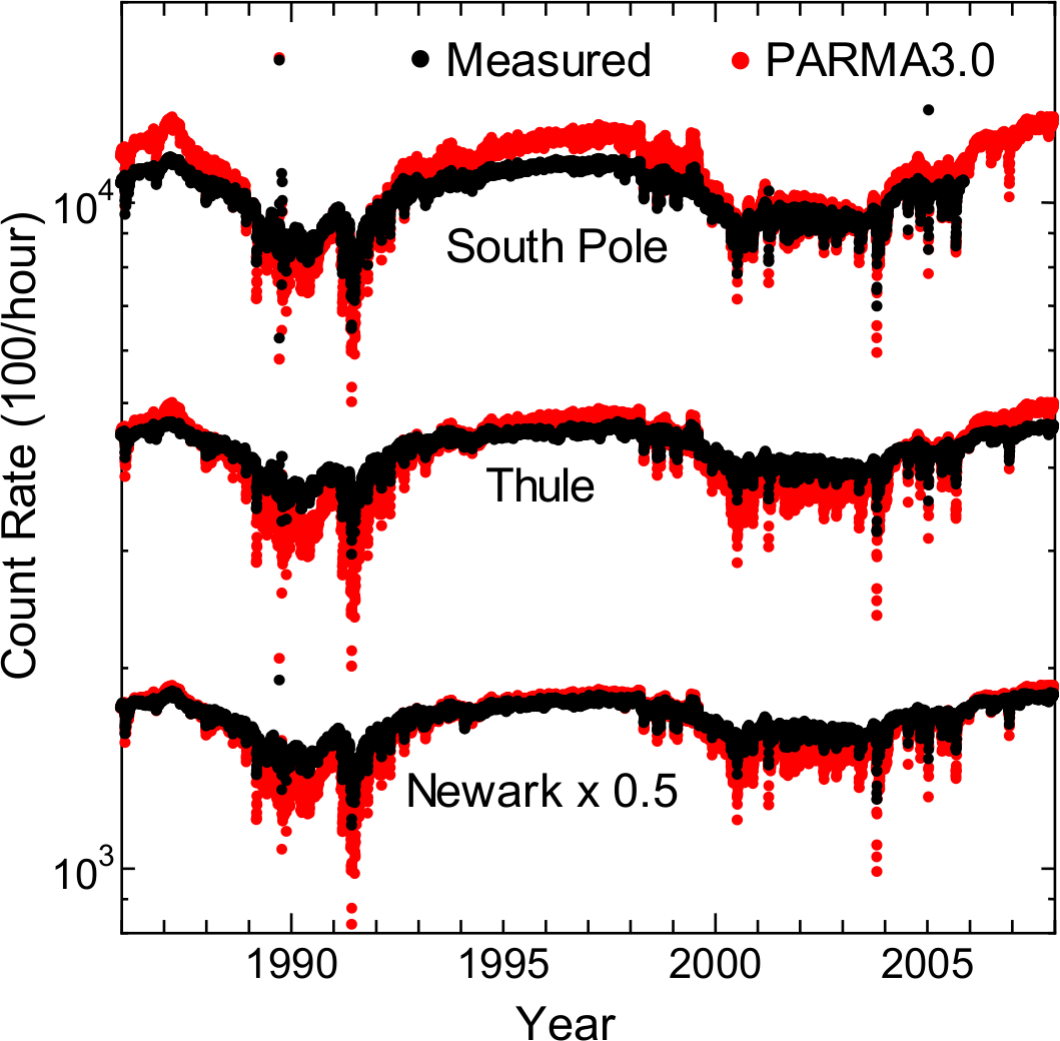
Count rates of neutron monitors in Thule, Newark, and South Pole calculated by PARMA3.0 compared with observed data [58].

The results of the comparison are shown in Fig 24. The agreement between the calculated and measured count rates is remarkably good in terms of absolute values. In contrast, the calculated results are more sensitive to solar modulation. In other words, PARMA3.0 slightly overestimated and underestimated the observations under the solar minimum and maximum conditions, respectively. The reason underlying this discrepancy is currently under investigation.

## Conclusions

Based on the results of the EAS simulation conducted using PHITS, we developed PARMA3.0, which facilitates instantaneous estimation of terrestrial cosmic ray fluxes nearly anytime and anywhere in the Earth’s atmosphere. The important features of PARMA3.0 in comparison to its previous version are (1) the maximum charge of the applicable ions has been increased from 2 (He) to 28 (Ni), (2) applicable altitude range has been extended from 20 km to the top of the atmosphere, and (3) minimum value of the applicable energy ranges, except for that of neutrons, was decreased from 1 MeV to 1 keV (per nucleon for ions). PARMA3.0 was verified and validated carefully using not only the results of the EAS simulation but also multiple sets of experimental data obtained under various global conditions. The new version of PARMA as well as the associated software EXPACS will be useful in diverse research areas such as geosciences, cosmic ray physics, and radiation research.
